# Comprehensive literature review of monkeypox

**DOI:** 10.1080/22221751.2022.2132882

**Published:** 2022-11-04

**Authors:** Ma’mon M. Hatmal, Mohammad A. I. Al-Hatamleh, Amin N. Olaimat, Suhana Ahmad, Hanan Hasan, Nurfatihah Azlyna Ahmad Suhaimi, Khaled A. Albakri, Anas Abedalbaset Alzyoud, Ramlah Kadir, Rohimah Mohamud

**Affiliations:** a Department of Medical Laboratory Sciences, Faculty of Applied Medical Sciences, The Hashemite University, Zarqa, Jordan; b Department of Immunology, School of Medical Sciences, Universiti Sains Malaysia, Kota Bharu, Malaysia; c Department of Clinical Nutrition and Dietetics, Faculty of Applied Medical Sciences, The Hashemite University, Zarqa, Jordan; d Department of Pathology, Microbiology and Forensic Medicine, School of Medicine, The University of Jordan, Amman, Jordan; e Faculty of Medicine, The Hashemite University, Zarqa, Jordan

**Keywords:** Monkeypox, MPX, MPXV, viral outbreak, emerging infectious diseases

## Abstract

The current outbreak of monkeypox (MPX) infection has emerged as a global matter of concern in the last few months. MPX is a zoonosis caused by the MPX virus (MPXV), which is one of the *Orthopoxvirus* species. Thus, it is similar to smallpox caused by the variola virus, and smallpox vaccines and drugs have been shown to be protective against MPX. Although MPX is not a new disease and is rarely fatal, the current multi-country MPX outbreak is unusual because it is occurring in countries that are not endemic for MPXV. In this work, we reviewed the extensive literature available on MPXV to summarize the available data on the major biological, clinical and epidemiological aspects of the virus and the important scientific findings. This review may be helpful in raising awareness of MPXV transmission, symptoms and signs, prevention and protective measures. It may also be of interest as a basis for performance of studies to further understand MPXV, with the goal of combating the current outbreak and boosting healthcare services and hygiene practices.

**Trial registration:** ClinicalTrials.gov identifier: NCT02977715..

**Trial registration:** ClinicalTrials.gov identifier: NCT03745131..

**Trial registration:** ClinicalTrials.gov identifier: NCT00728689..

**Trial registration:** ClinicalTrials.gov identifier: NCT02080767..

## Introduction

Monkeypox virus (MPXV) is a member of a subset of the *Poxviridae* family called *Orthopoxvirus*. This virus causes infection with clinical presentation resembling smallpox (SPX), which is caused by infection with the variola virus (VARV). MPXV was first isolated in 1958 from laboratory monkeys with a pox-like disease in a Copenhagen research facility in Denmark [1,2]. Genomic studies have characterized MPXV into Central African/Congo Basin and West Africa clades with differential epidemiology and clinical manifestations [3]. Most MPXV outbreaks outside Africa come from the West Africa clades with less severe disease and primary infection [4].

Since MPX infection has a similar presentation to many pox-like diseases, diagnosis based on clinical observations alone is insufficient. Thus, real-time PCR is used to distinguish the two MPXV clades from other orthopoxviruses [5,6]. Coincident immunity against MPXV has been achieved through SPX vaccination due to shared genetic and antigenic properties. Since SPX eradication and hence vaccine cessation, waning herd immunity to orthopoxviruses has created an immunologically naïve population which, along with several other factors, has led to resurgence of MPXV [7].

Since the beginning of 2022, cases of MPX from several regions have been reported to the World Health Organization (WHO), indicating an alarming re-emergence of MPX. On 13 May 2022, the WHO confirmed a multi-country MPXV outbreak in Africa and non-endemic countries worldwide, especially in Europe. By 13 June 2022, the Pan American Health Organization (PAHO) and WHO (PAHO/WHO) had recorded a total of 1423 confirmed cases of MPX in 31 non-endemic countries with no deaths. Around 87% of these cases were reported in 23 countries in the European region [8]. Concern has grown about the ongoing MPX outbreak as there is a shortage of new reports, and this has led to proliferation of misleading information. The goal of this review is to examine the origin of MPX and its evolution, transmission, pathogenesis, diagnosis, epidemiology, host immunity, treatment and prevention.

## Molecular basis of MPXV activity

**Figure 1.**
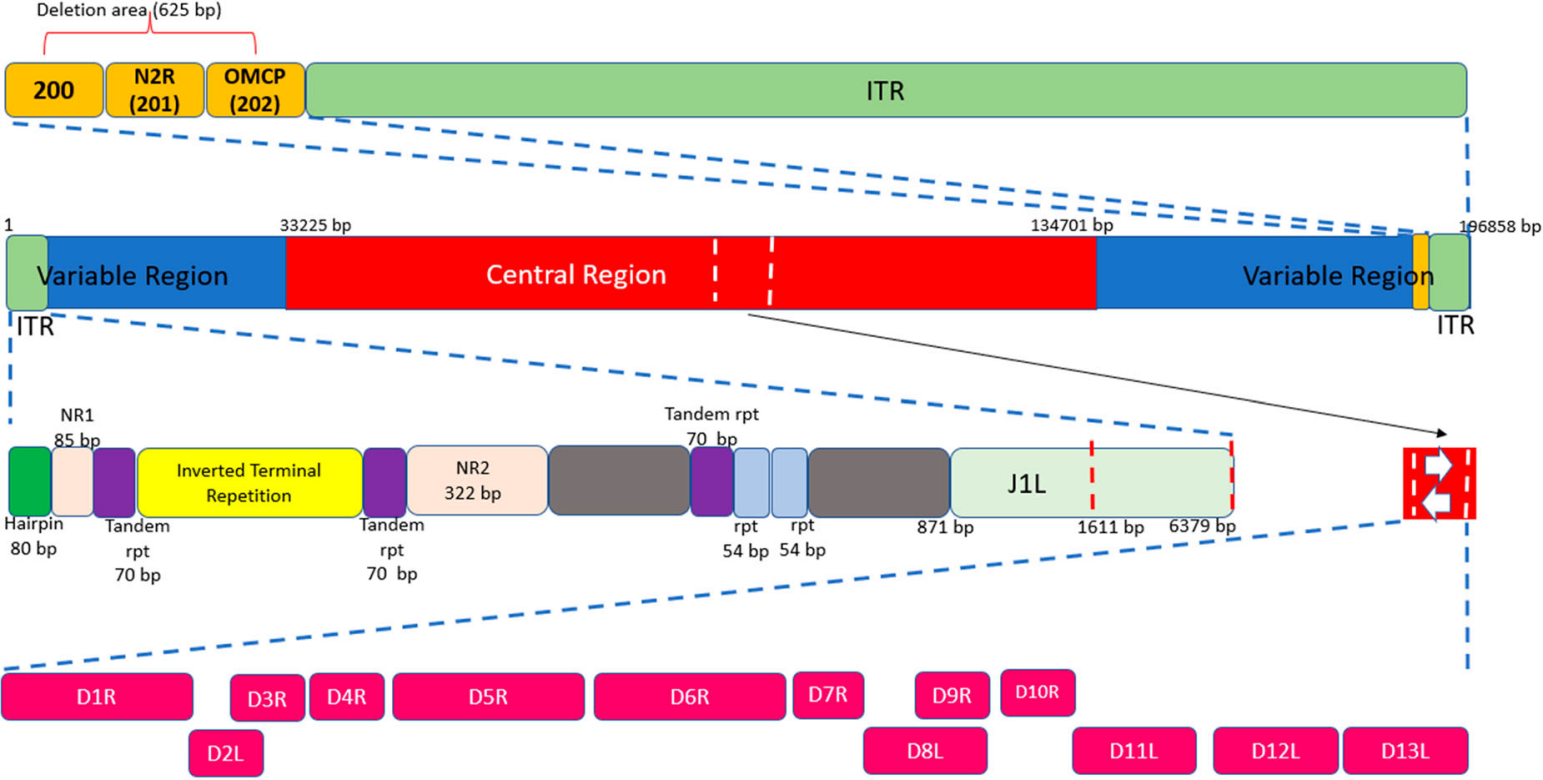
General structure of the MPXV genome. The genome is made up of double-stranded linear DNA (approximately 197 kb), primarily composed of hairpin loops, some open reading frames (ORFs), and tandem repeats, while the ITRs are made up of tandem repeats, hairpin loops, and some ORFs [13]. The ends of the genome form direct repeats called ITRs, and the genome has a terminal hairpin loop (no free ends). Most of the essential genes are located in the central part of the genome, and there are ∼250 genes in the genome [14]. The upper box reveals a 625-bp deletion directly upstream of the right ITR (red box), which completely removes MPV-Z-N2R (locus 201) and truncates OMCP (MPV-Z-N3R, locus 202). The central part contains the following genes: D1R: large subunit of mRNA capping enzyme, D2R and D3R: internal structural proteins of intracellular mature virions (IMVs), D4R: viral DNA glycosylase, D5R: ATPase, D6R: subunit of early transcription protein, D7R: subunit of RNA polymerase, D8R: membrane protein of IMV, D9R, D10R, D11R: nucleotide triphosphate phosphorylate, D12R: small subunit of mRNA capping enzyme, and D13R: core protein of IMV [14].

The MPXV has a double-stranded DNA genome of 196,858 base pairs (bp) with around 200 genes [9]. As an orthopoxvirus, its genome contains two telomeres composed of identical but oppositely-oriented sequences of short tandem repeats [10]. This region of inverted terminal repeats (ITRs) makes up around 3% (6379 bp) of the MPXV genome and is involved in the replication and encapsidation of the genome [11,12]. Further details of the genomic organization of MPXV are shown in Figure 1.

MPXV encodes all transcription and replication enzymes needed for the viral genome [14]. It has been hypothesized that the progressive loss of genes not essential for human pathogenesis led to the emergence of a highly adapted virus that causes serious disease and is capable of efficient and rapid human-to-human transmission [15,16]. According to a study by Elde and colleagues, gene copy number variation may be a key element in regulating virus fitness [17]. Among the MPXV alignments, polymorphism in the noncoding region of the ITR with 12 variants was detected. Four of the 23 (17.4%) whole genome sequences displayed significant genomic instability just upstream of the right ITR. A 625-bp deletion between bases 189,820 and 190,444 was present in a collection of samples (genome locations based on MPXV-COG 2003 358). MPV-Z-N2R and the first 103 bp of the genus *Orthopoxvirus* major histocompatibility complex class I-like protein (OMCP) are both removed by this deletion. The function of MPV-Z-N2R is unknown, as neither the VARV nor the West African MPXV genomes contain any similar genes. OMCP is a secreted protein that binds to NKG2D and prevents natural killer cells from destroying infected cells [16].

Although MPXV is a DNA virus, its entire lifecycle occurs in the cytoplasm of infected cells, in which a variety of proteins needed for the replication machinery are encoded from open reading frames (ORFs) of the MPXV genome (Table 1). These ORFs have more than 90% sequence identity with those of other orthopoxviruses. The majority of species- and strain-specific differences between orthopoxviruses are in the left and right terminal regions [9].

**Table 1.** List of the most important ORFs in the MPXV genome and their functions.

ORF	Size	Functions
*D5R*	242	Zinc-binding, virulence factor and inhibition of UV-induced apoptosis
*P1L*	117	Secretion of virulence factor
*C2L*	375	Synthesis of serine protease inhibitor-like (SPI-3) and prevents cell fusion
*C8L*	151	Deoxyuridine triphosphatase production
*C16L*	439	Encoding serine/threonine protein kinase 2 and regulation of virion morphogenesis
*C23R*	101	Virion core DNA binding phosphoprotein
*F1L*	479	Poly(A) polymerase and catalytic subunit
*F3L*	153	dsRNA binding inhibits dsRNA-dependent protein kinase and 2-5A-synthetase
*F4L*	259	RNA polymerase, 30-kDa subunit and intermediate stage transcription factor
*F8L*	1006	DNA polymerase
*Q2L*	108	Virion-associated glutaredoxin
*I1L*	312	Virosomal protein needed for virus multiplication
*I3L*	269	ssDNA-binding *P*-protein interacts with R2 subunit of ribonucleotide reductase
*I4L*	771	ssDNA-binding *P*-protein interacts with R1 subunit of ribonucleotide reductase
*I7L*	423	Virion core protein, DNA topoisomerase II homologue from
*G4L*	124	Virion-associated glutaredoxin, required for disulphide bonds and assembly
*H5R*	213	Virosome-associated, late gene transcription factor, VLTF-4, Ca2^+^-binding motif
*E5R*	785	Nucleic acid-independent nucleoside triphosphatase, required for DNA replication
*A11L*	891	Major virion core protein p4a
*A13L*	190	Virion core protein
*A19R*	492	DNA helicase, post replicative negative transcription elongation factor
*A34L*	300	DNA packaging into virion and NTP-binding motif A
*A50R*	554	DNA ligase

Source: Adapted from Shchelkunov et al. [9].

Some viral proteins have been found to be essential components of MPX. These proteins are classified into three categories: (1) viral entry proteins that facilitate MPXV entry into host cells through receptor binding and membrane fusion; (2) viral proteins that facilitate release of MPXV copies from host cells; and (3) essential proteins for modulation of the host cell and immune modulation. These proteins are summarized in Table 2 and their roles in host cells are further discussed in the next sections.

**Table 2.** List of known MPXV proteins, their encoding genes and host target proteins.

Gene	Protein	Host target proteins
Entry proteins		
*M1R*	Protein L1 (virion membrane protein)	Probably binds to host cell entry receptors
*E8L*	E8L (cell surface-binding protein)	Cell surface chondroitin sulphate proteoglycans (CSPG)
*H3L*	H3L (envelope protein)	Cell surface heparan sulphate (HS)
Exit proteins		
*A38R*	IEV (transmembrane phosphoprotein)	NCK adaptor protein 1 (NCK1), kinesin light chain 1 (KLC1), intersectin-1 (ITSN1) and epidermal growth factor receptor substrate 15 (EPS15)
*C23R*	Core phosphoprotein F17	Rapamycin-insensitive companion of mTOR (RICTOR) and regulatory-associated protein of mTOR (RPTOR)
*C18L*	Protein F12	Kinesin light chain 2 (KLC2)
Immunomodulatory proteins		
*J3R*	Chemokine binding protein	CC and CXC chemokines
*J2L*	Cytokine response-modifying protein B	Tumour necrosis factor-alpha (TNF-α), TNF-β, CC motif chemokine ligand 28 (CCL28), CCL25, CXC motif chemokine ligand 12 (CXCL12), CXCL13 and CXCL14
*D9L*	Ankyrin repeat domain containing protein CP77 (type I interferon (IFN) evasion protein)	Cullin-1 (CUL1) in the SKP1-CUL1-F-Box (SCF) complex
*F3L*	RNA-binding protein E3	Interferon-stimulated gene 15 (ISG15), eukaryotic translation initiation factor 2 alpha kinase 2 (EIF2AK2)/protein kinase R (PKR) and Z-DNA binding protein 1 (ZBP1)
*H1L*	Dual specificity protein phosphatase H1	Signal transducer and activator of transcription 1 (STAT1)
*D3R*	EGFR binding protein (MPXgp006)	Epidermal growth factor receptor (EGFR)
*D11L*	Protein C6	TRAF family member associated NF-κB activator (TANK), TANK-binding kinase 1-binding protein 1 (TBKBP1), 5-azacytidine-induced protein 2 (AZI2) and STAT2
*C7L*	Protein F1	Bcl-2-like protein 11 (BCL2L11), NLR family pyrin domain containing 1 (NLRP1) and Bcl-2 homologous antagonist/killer (BAK)
*B16R*	Soluble IFN-alpha receptor	IFN-α
*C1L*	IFN antagonist K1L	IFN
*B13R*	Protein B13	IκB kinase β (IKKβ)
*B9R*	Soluble IFN-γ receptor B8	IFN-γ
*P1L*	Protein N1	Bcl-2-associated agonist of cell death (BAD) and Bcl-2-associated X protein (BAX)
*C6R*	Protein K7	DEAD-box helicase 3 X-linked (DDX3X)
*A37R*	MHC modulating protein	Major histocompatibility complex (MHC) class II
*A41L*	Protein A41 (chemokine binding protein)	CCL21, CCL25, CCL26 and CCL28
*A47R*	TLR inactivating protein (MPXgp157)	Myeloid differentiation factor-88 (MyD88) adaptor-like and TIR-domain containing adapter-inducing interferon-β (TRIF)-related adaptor molecule (TRAM)

Data were collected from https://viralzone.expasy.org/9976 and https://www.uniprot.org/.

Like other orthopoxviruses, the pattern of MPXV gene distribution has the genes that encode for housekeeping functions conserved and clustered in the central region of the genome (Figure 1), whereas those that encode for proteins involved in virus-host interactions are less conserved and located in the terminal regions [13,18–23]. It has been hypothesized that MPXV is a direct ancestor of VARV based on the similarity in the clinical manifestation of the two diseases [24–26]. Later studies confirmed this hypothesis by detecting high similarity in genetic material between the two viruses using genomic restriction endonuclease maps [27,28] and nucleotide sequencing [29,30]. However, other studies have postulated the independent evolution of the viruses [27,31]. Whole genome sequencing has shown that MPXV is not a direct ancestor nor a direct descendant of VARV [32].

The genetic diversity between the West African and Congo Basin clades has been documented in several studies of the evolutionary relationships between the clades [33]. The clades are 99.4% identical at the protein level, but include several functionally unique genes, non-functional ORF regions and additional ORFs [33].

During the multi-country 2022 outbreak, several preliminary phylogenetic analyses of MPXV genomes were performed from samples collected in Portugal, Belgium, France, Germany, the Netherlands, Italy, Spain, Slovenia and Brazil. The data confirmed that the West African MPXV genotype is central to the ongoing outbreak. A total of 117 MPXV sequences were identified up to 24 June 2022 (Supplemental Table 1) using the NCBI database related to the current outbreak of MPXV outside the endemic area. Notably, detection of MPXV was correlated with individuals who had returned from the Canary Islands [34], Slovenia [35], Italy [36] and France [37].

The sequences from Slovenia were from two patients who presented with anogenital skin lesions, swollen inguinal nodes and malaise. Skin sampling was used to isolate the MPXV genome from the French sample. Nasopharyngeal swabs, lesion crust and vesicles were used as viral genetic material harvested after one week-onset of mild symptoms, including fever and odynophagia, from the sample from the Canaries. Sequences were also obtained from a 30-year-old male who presented in Belgium with perianal papules and a 1-cm painful inguinal adenopathy bilaterally after travelling to Lisbon, Portugal [38]. A 41-year-old male patient was diagnosed with MPX after a trip to Portugal, Spain and Brazil [39].

The greatest number of sequences (50) was from a study in Germany, in which whole genome sequencing was used for samples from a 26-year-old patient who presented with acute symptoms of orthopoxviral infection [40]. A further 28 sequences were detected using the paired-end sequencing technique in Portugal [41].

Most MPXV sequence isolates have been obtained from male patients from Belgium [38], Portugal [42], Italy [36,43], Brazil [39] and Spain [44]. Infection with human immunodeficiency virus (HIV) was reported in two cases: a 31-year-old Spanish man [44] and a 39-year-old Italian man who had HIV infection with a history of unprotected sex with male partners [36].

A number of novel single nucleotide polymorphisms (SNPs) among newly-detected MPXV sequences have been identified during the 2022 outbreak, including 46 SNPs in newly-discovered MPXV sequences from Spain, in comparison with genomes from the 2018/2019 outbreak [34]. Six SNPs were identified between the two draft genomes from Madrid [44] and 6 from Italian sequences, in comparison to other MPXV genomes detected during the 2022 outbreak.

Microevolution of MPXV may explain the newly-detected clusters of viral genomes during the 2022 outbreak caused by the emergence of 7 SNPs leading to further subclusters and sub-branching from the common ancestor [41]. A frameshift deletion of 913 bp in the viral genome has been reported in two sequences from Portugal [41]. The effect of the number of SNPs detected in this genome compared to those isolated in the UK during the 2018–2019 outbreak led to synonymous, missense, stop-gained and intergenic variants [41]. These microevolution events enhance the evidence of human-to-human transmission of MPXV strains evolved from the West African ancestor of the MPXV currently detected outside the endemic area [16]. The MPXV isolated from the 2022 outbreak seems to have more mutations, but many of these newly acquired mutations have unclear function and significance [45]. These mutations could be the underlying cause of the sudden emergence of MPX cases in non-endemic areas. However, this can be ruled out because DNA viruses have lower per-site mutation rates due to the extensive interactions between viral DNA genomes and cellular pathways that detect and repair DNA damage, compared to RNA viruses (e.g. HIV and SARS-CoV-2) [46]. Therefore, further studies are required to determine the mechanism of action of these mutations.

## Pathogenesis

The pathogenesis and mechanism of action of MPXV are similar to those of VARV and *Vaccinia* virus (VACV) [47]. MPXV, like other poxviruses, probably infects a wide range of mammalian cells without the need for specific host receptors and molecules for cell entry and replication [48]. The infection process begins with binding and entry of the extracellular enveloped virus (EEV) virions into the host cell through interactions of MPXV surface proteins with primary attachment receptors (glycosaminoglycans) on the cellular membrane of host cells [49]. Information on specific MPXV proteins involved in host cell entry and the receptors on host cells is currently lacking, but three proteins have been identified as viral entry proteins that may facilitate MPXV entry into host cells through receptor binding and membrane fusion. The first is protein L1, a virus membrane protein that probably binds to host cell entry receptors. The specific roles of protein L1 during MPXV entry are unconfirmed, but studies on VACV show that this envelope protein binds to the cell surface through the entry/fusion complex (EFC) and is required for merging the virus membrane to the host cell membrane during viral penetration [50–52]. E8L is another MPXV cell surface-binding protein that is suggested to bind to host cell surface chondroitin sulphate proteoglycans (CSPG) and mediate adsorption of intracellular mature virus (IMV) virions to cells [53]. The MPXV envelope protein H3L has also been studied in *in-vitro* and *in-vivo* on VARV, indicating important roles for this protein in virus adsorption to cell surface heparan sulphate and IMV morphogenesis [54]. Despite the variability in surface glycoproteins and the number of wrapping membranes between the IMV and EEV virions [55], IMVs that exit infected cells through budding can also penetrate the cellular membrane and infect other host cells, but less efficiently than EEV [56].

**Figure 2.**
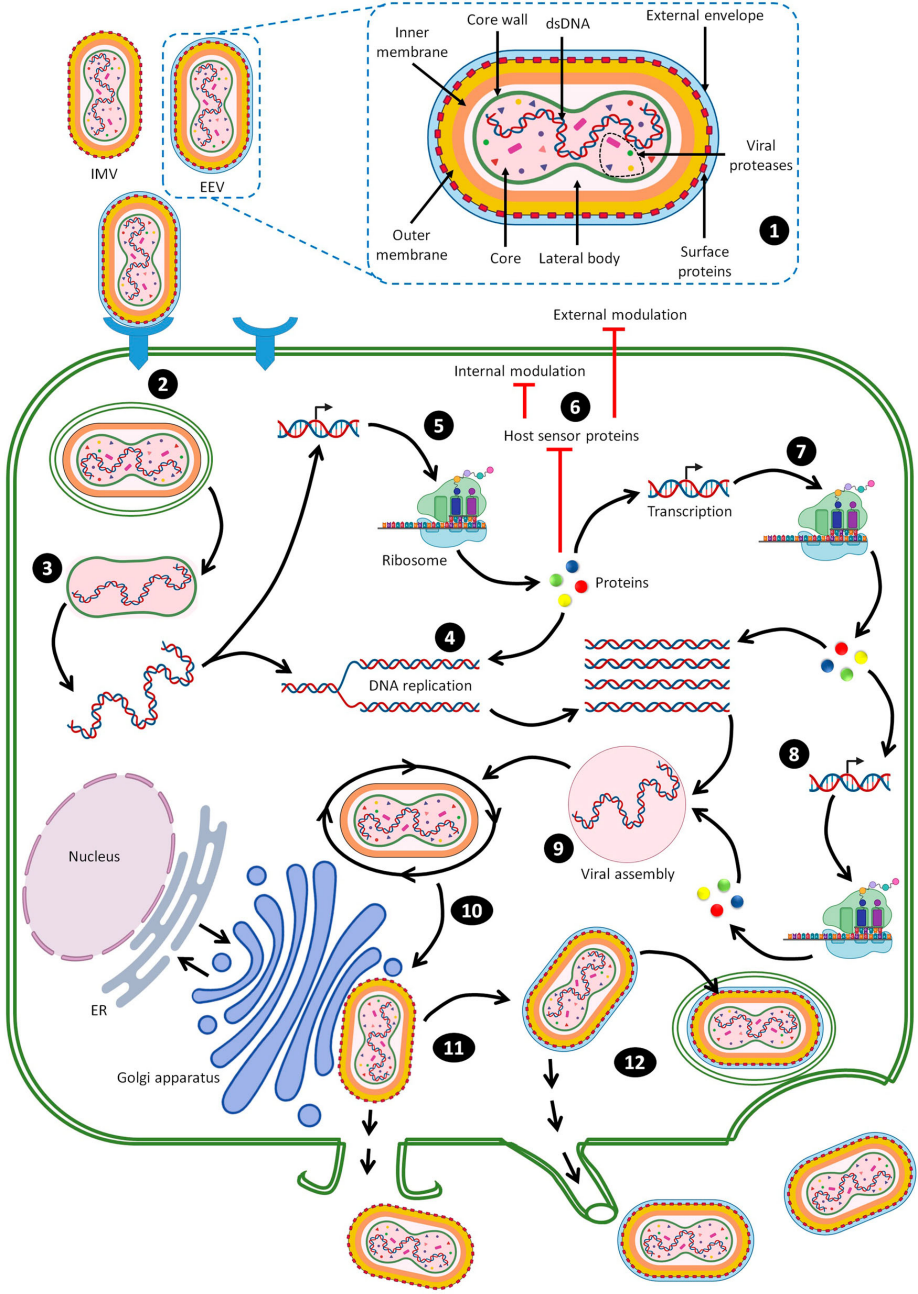
Steps of MPXV entry into host cells [13,14,59–62]. (1) Schematic of the structure of MPXV. (2) Both the EEV and IMV virions penetrate the host membrane by binding and macropinocytosis. MPXV virions use glycosaminoglycans as host receptors. (3) After the internal virion components enter the cytoplasm, core uncoating occurs and this process leads to delivery of the MPXV genome and accessory proteins to the cytosol. (4) The released MPXV genome is used as a template for DNA replication. (5) Early viral DNA transcription followed by translation into the host ribosome occurs to encode essential proteins. Early proteins aid in DNA replication. (6) These proteins interact with host sensor proteins resulting in internal and external modulations. The major intracellular modulations include prevention of viral genome detection, induction of cell cycle arrest, apoptosis inhibition, inhibition of the antiviral system and modulation of some host cellular signalling pathways. Early proteins play essential extracellular roles as immunomodulatory agents and as growth factor-like domains that stimulate onset of mitosis in neighbouring cells. (7) Early proteins are used in production of intermediate proteins. (8) These proteins are involved in late transcription and translation processes and aid in DNA replication. (9) Late proteins are essential components for viral assembly. (10) Viral morphogenesis occurs by formation of inner tubular nucleocapsid structure folding and assembly of viral glycoproteins to generate MV virions. (11) Except those released via infected cell lysis, MV virions transit to the Golgi apparatus along microtubules for double membrane wrapping. (12) The resulting EEV virions exit the infected cell by two routes: by the actin tail assembly, which provides enough force to propel the virions out of the cell or by budding from a cellular membrane (Created with BioRender.com).

After fusion of MPXV EEV or IMV with the cellular membrane, the internal virion components are spontaneously uncoated with loss of viral membranes and enter the host cytoplasm [57]. All poxviruses replicate their nucleic acid exclusively in the cytoplasm and encode proteins that facilitate genome replication and gene expression [58]. The cytoplasmic replication cycle of MPXV is a complex sequence of events that needs further investigation, but the intracellular cycle of MPXV can be visualized based on understanding of VACV replication as the best-studied poxvirus (Figure 2).

The first attempt at understanding the pathogenesis of MPXV was made in 1969 by Wenner et al. [63]. Cynomolgus monkeys were infected with MPXV intramuscularly and the virus started to multiply in local cellular components at the injection site. In addition to detection of MPXV at the site of inoculation, an intense inflammatory immunoresponse is seen in cell necrosis, phagocytosis, vasculitis and local replication of MPXV [63]. Primary viremia then developed based on detection of the virus in regional lymphatic and vascular channels. MPXV is transported in lymph to regional lymph nodes and very likely in blood to the spleen, tonsils and bone marrow. These organs, among others, comprise secondary sites of virus multiplication and with further release of the virus, there is a consistently measurable level of viremia. At this stage, it is likely that the virus is transported to tertiary target organs, including the skin and testes, resulting in clinically recognizable disease.

The difficulty in understanding the pathogenesis of MPXV is due to the lack of ideal animal models with routes of MPXV transmission similar to those in humans, similar pathways of pathogenesis, and similar rates of infection, morbidity and mortality [64,65]. The main challenge is the resistance and non-infectivity of the virus in commonly used animal models, such as guinea pigs and golden hamsters [64,66]. However, *in-vitro* investigations of the kinetics of the poxvirus replication cycle using different cell lines have indicated similarity of MPXV with VACV and VARV in the production of viral antigens, patterns of cytopathological change and formation of inclusion bodies, and release of new virions from host cells [67–71]. Despite some differences among poxviruses based on the type of cell culture lineage and cell growth conditions, a better understanding of MPXV has emerged based on VACV studies.

The first step in MPXV replication following inoculation is virion attachment to the target cells, which has been investigated *in vitro* using rhesus monkey kidney cells or kappa cell lines, in which up to 85% of virion particles are found to attach within 2 h [72]. Synthesis of messenger RNA has a vital role in the uncoating process [47].

Using 2 plaque-forming units (PFU)/cell to infect CV-1 cells reveals a 6-h period of partial eclipse, presumably representing the period of attachment, uncoating and synthesis of the earliest virions. The pattern of increase of cell-free virus follows closely with that of cell-associated virus, with a lag of 3 or 4 h between intracellular maturation and extracellular release [72,73]. The newly synthesized virus releases from kappa cells at a rate of 1% [72] and from CV-1 cells at 10% [73]. MPXV antigens can be detected in nuclear region or long cellular bridges of infected cells using cytoplasmic immunofluorescence [73].

Cytopathic effects of MPXV have been observed in primary and secondary lines of kidney cells derived from rhesus, cynomolgus and African green monkeys [72–75], bovine, rabbit and guinea pig kidneys, mouse liver cells [33,72] and human-derived amnionic and lung fibroblasts [75,76]. Cytopathic effects have not been reported for all cell lines, but a few lines in HeLa cells, chicken embryo and other cell cultures have shown these effects [72,76,77]. Granulation, rounding up and cellular condensation have been reported as cytopathic effects caused by MPXV, and monolayer cells detached from the side of the glass, leaving microscopic visible “holes.” Affected cells in monkey kidney and human amnion cell cultures are interconnected by thread-like syncytial elongations, but such cellular bridges are not apparent in HeLa cells [75].

Depending on the size of the inoculum, CPE- of MPXV-infected CV-1 cells (a continuous line of African green monkey kidney cells) may be observed as early as 8 h or as late as 10 days or more [73]. When a suspension of pustular material from infected monkeys is inoculated into such tissue cultures, the CPE usually develops in 2–3 days. Complete destruction occurs after 5 days of incubation [75]. In tissue cultures, the infectivity titres of most passage fluids vary between 10^−4^ and 10^−6^ for the 50% tissue culture infective dose (TCID_50_) [73,75]. The physical characteristics of CPE produced by MPXV in monkey kidney-cultured cells cannot be distinguished from those of VARV [77] or VACV [73].

In addition to cytopathic effects, plaque formation with MPXV has been detected in various cell culture lines [72,73,78]. The plaque formation assay is a quantitative method, in which monolayers of monkey kidney cells infected with MPXV are allowed to overgrow and then stained using neutral red to demonstrate well-defined plaques of 2–3 mm in diameter [72,73,79]. Previously, MPXV was differentiated from VARV by the smaller size of the plaques [78] and by the ability of MPXV to form plaques in chicken embryo fibroblasts [78,80].

As viruses are intracellular and host-dependent microorganisms [81], survival inside infected host cells is critical for virus propagation, and this is based on manipulation of host cell signalling pathways. This manipulation enhances the viral replication cycle and determines disease outcomes [82], mainly by targeting cell growth and immunoregulation [83]. Thus, orthopoxviruses can inhibit cell apoptosis and the antiviral host defence, and exploit the host cell machinery [84].

## MPXV immunomodulatory proteins and related immune responses

As shown in Table 2, a variety of MPXV proteins are implicated in host immunomodulation after being encoded in host cells. The chemokine binding protein encoded by the MPXV *J3R* gene binds to CC and CXC chemokines with high affinity, regulating leukocyte trafficking to tissues infected with MPXV and thus reducing viral virulence and inflammatory response [85,86]. Protein A41 is another chemokine binding protein that is encoded by *A41L* and targets the CC motif chemokine ligands CCL21, CCL25, CCL26 and CCL28. Bahar et al. [87] suggested that A41 forms sufficient interactions with these chemokines to prevent chemokine-glycosaminoglycan interactions at the cell surface, thereby destroying the chemokine concentration gradient and ultimately resulting in decreased neutrophil migration in tissues infected with MPXV [87,88]. The MPXV-encoded cytokine response-modifying protein B (CrmB) helps the virus to evade host immune defence by binding to host tumour necrosis factor (TNF) as a soluble decoy TNF receptor (TNFR) [89]. Thanks to its C-terminal domain, CrmB also binds to CCL28, CCL25, CXC motif chemokine ligand 12 (CXCL12), CXCL13 and CXCL14, with binding affinities comparable to those of TNF [89].

MPXV also encodes Ankyrin repeat domain containing protein CP77, which plays an early role in evading the antiviral state induced by type I interferon (IFN) by binding to cullin-1 (CUL1) in the SKP1-CUL1-F-Box (SCF) complex [90]. The SCF is an ubiquitin-protein ligase complex. It has been suggested that, following C-terminal phosphorylation, IFN regulatory factor 3 (IRF3) is recognized by CUL1, which is part of the SCF complex [91]. This leads to its polyubiquitination and targeting of the proteasome, indicating a significant role for the SCF complex in controlling IRF-3 stability [91]. IRF3 controls multiple IFN-inducing intracellular pathways that are triggered by RNA and DNA sensors [92]. Two MPXV genes, *B16R* and *B9R*, encode proteins that mimic the soluble IFN-α and IFN-γ receptors, respectively. These two proteins bind to IFN-α and IFN-γ to block the functions of IFNs, thereby inhibiting defences against MPX infection [93,94]. Also, the MPXV K7 protein binds to DEAD-box helicase 3 (DDX3) and inhibits *IFN-β* promoter induction [95]. DDX3 is a multifunctional protein involved in RNA metabolism and plays an essential role in key cellular biogenesis processes [96]. A recent study showed that DDX3 has a critical role in promoting *IFN-β* transcription formed by antiviral signalling by enhancing IRF-3/p300 holocomplex binding to the *IFN-β* promoter [97].

IFN antagonist K1L is another protein encoded by MPXV that inhibits the IFN-induced antiviral system [98]. K1L may not block IFN signalling pathways directly, but it prevents acetylation of the p65/RelA subunit of nuclear factor kappa B (NF-κB) [99]. NF-κB signalling is involved in regulation of major immune functions, especially by inducing antiviral genes such as IFN and IFN-stimulated genes (ISGs) [100]. MPXV also encodes the B13 protein that binds IκB kinase β (IKKβ), which contributes to IκBα phosphorylation and NF-κB activation [101], ultimately resulting in blocking the NF-κB signalling pathway by inhibiting IKKβ dimer trans-autophosphorylation as part of the activation mechanism [102].

MPXV encodes the dual specificity protein phosphatase H1 (H1L), which is involved in viral replication [103] and also has a role in immune evasion by blocking IFN-induced antiviral immune responses by dephosphorylating signal transducer and activator of transcription 1 (STAT1). It has been suggested that H1L can also block expression of STAT1-dependent and STAT1-independent genes [104]. STAT1 has an essential role in controlling expression of human IFNs and thus the severity of viral infections [105]. STAT1 is also involved in immunoglobulin (Ig) class switch recombination (CSR) and in production of memory B cells that contribute to tissue-resident humoral immunological memory by controlling the IgG response against viral reinfection [106].

MPXV protein C6 works as a viral immunomodulator by binding to the transactivation domain STAT2. This association decreases STAT2 phosphorylation and results in blocking of IFN signalling pathways [107] and forms an integral part of the transcriptional responses to IFNs [108]. MPXV protein C6 also binds TRAF family member associated NF-κB activator (TANK) and inhibits IFN regulatory factors 3 and 7 (IRF3 and IRF7) [109]. IFRs are transcription factors that play crucial roles in several innate and adaptive immune responses, including the antiviral state and regulation of immune cell differentiation. These proteins are key regulators of induction of IFN gene expression downstream of pathogen recognition receptors (PRRs), such as Toll-like receptors (TLRs), which recognize viral nucleic acid [92]. The nature of the signalling complexes formed on regulation by IRFs leads to further targets for MPXV protein C6. Thus, C6 binds to TANK-binding kinase 1-binding protein 1 (TBKBP1), which is an adaptor protein that binds to TBK1 and inhibits activation of IRF3 and IRF7 [109]. Since TBKBP1 is part of the interaction network in the TNF and NF-κB pathway [110], protein C6 may contribute to MPXV immune evasion via other cellular pathways.

MPXV A47R protein also inhibits the TLR signalling pathway by targeting myeloid differentiation factor-88 (MyD88) and TIR-domain containing adapter-inducing interferon-β (TRIF)-related adaptor molecule (TRAM), which are well-known as adaptors for inflammatory signalling pathways downstream of members of the TLR family [111]. MPXV evades host immune defence using the A37R protein, which targets MHC class II and suppresses the MHC class II antigen presentation pathway by affecting the stability or intracellular sorting of these proteins [112]. Class II MHC proteins facilitate the presentation of viral proteins found in the cytoplasm and exocytic compartments after macroautophagy by antigen-presenting cells (APCs) [113].

To counteract the viral inflammatory response, MPXV encodes RNA-binding protein E3, which inhibits ISG15 [114] and targets eukaryotic translation initiation factor 2 alpha kinase 2 (EIF2AK2)/protein kinase R (PKR) [114] as a crucial enzyme for regulation of the integrity of newly synthesized IFN mRNA [115]. E3 also targets Z-DNA binding protein 1 (ZBP1), also known as DNA-dependent activator of IFN regulatory factors (DAI) [116], which works as a cytoplasmic DNA sensor and functions in the development of immune responses [117]. ZBP1 plays a crucial role in controlling virus replication, and deletion of ZBP1 is significantly associated with severe viral infections [117,118]. ZBP1 binds to the receptor-interacting protein kinase 3 (RIP3) to form a complex that mediates virus-induced programmed necrosis [119].

MPXV uses protein F1 in targeting the nucleotide-binding domain (NBD) and leucine-rich repeat (LRR) receptor (NLR) proteins and binds NLR family pyrin domain containing 1 (NLRP1) [120]. NLRP1 is involved in the formation of inflammasomes as important cytosolic multiprotein oligomers of the innate immune system [121]. Caspase-1 cysteine protease activation by inflammasomes is a crucial immune response to viral infections, as it stimulates production of IL-1β, IL-18 and high mobility group box 1 (HMGB1) protein to initiate pronounced inflammatory responses. Caspase-1 also triggers pyroptosis of host cells to eliminate the virus [122]. It may thus be beneficial to MPXV to hijack the inflammasome machinery and inhibit caspase-1 activation by targeting NLRP1 with protein F1.

MPXV proteins F1 and N1 target the B-cell lymphoma 2 (Bcl-2) family of proteins that control cellular apoptosis. Through the proapoptotic Bcl-2 homology 3 (BH3) domain, F1 binds host pro-apoptotic Bcl-2-like protein 11 (BCL2L11) and Bcl-2 homologous antagonist/killer (BAK) [123], while N1 binds Bcl-2-associated agonist of cell death (BAD) and Bcl-2-associated X protein (BAX) [124,125]. Since BCL2L11, BAK, BAD and BAX are all involved in altering apoptosis and autophagy by elimination of BH3-only proteins [126–128], both F1 and N1 may have anti-apoptotic roles in MPX infection. Bcl-2 has also emerged as a regulator of innate immune responses [129].

MPXV encodes protein MPXgp006 containing an epidermal growth factor (EGF)-like domain that targets the EGF receptor (EGFR). This domain binds an ErbB protein containing four receptor tyrosine kinases and is structurally related to EGFR [130]. The EGFR signalling pathway is among the most crucial in mammalian cells and involves complex processes that regulate a wide range of essential cellular functions such as apoptosis, differentiation and proliferation. This pathway also has a role in regulating intercellular communication [131]. Thus, MPXV relies on EGFR-regulated pathways to invade host cells and turn them into virus-making factories [132]. A previous study on VACV confirmed that poxviruses hijack EGFR-induced cell motility to enhance efficient virus spread and pathogenesis [133].

**Figure 3.**
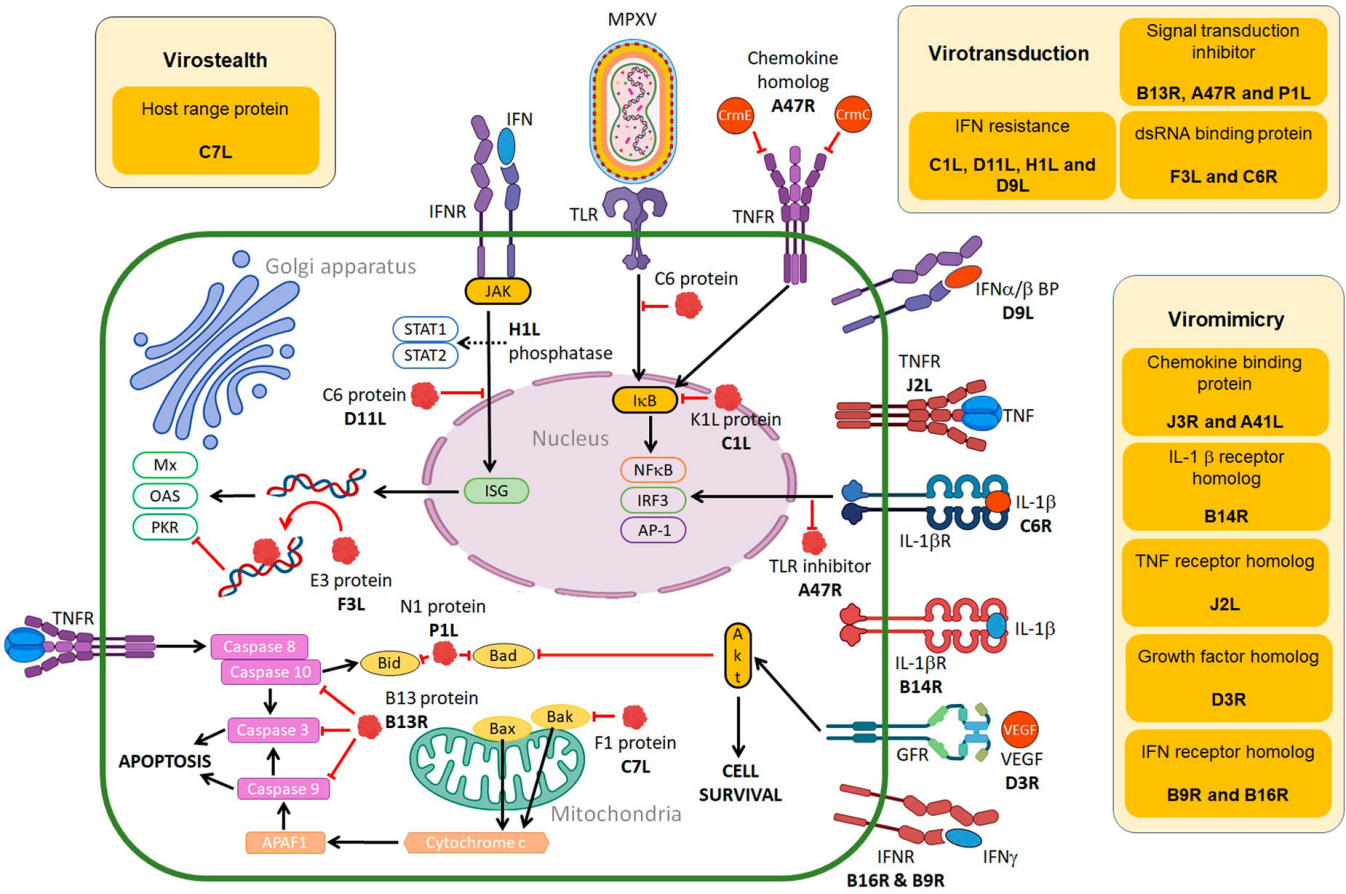
MPXV proteins (red) that participate in virostealth, viromimicry and virotransduction are responsible for immune evasion mechanisms of MPX infection [134–137]. In viromimicry, MPXV mimics host receptors that inhibit binding of IFN, IL-1β and TNF as well as MPXV-encoded chemokines and growth factors. In virotransduction, several antiviral pathways including IFN, NF-κB, IRF3 and apoptosis are interfered with by intracellular MPXV-encoded proteins to inhibit their functions. Virostealth is achieved with F1, an anti-apoptotic host range protein that helps with viral replication and the spread of MPX infection (Created with BioRender.com).

MPXV immunomodulatory proteins can be subdivided by function into three distinct categories: virostealth, virotransduction and viromimicry (Figure 3). The virostealth proteins act intracellularly, reducing detection of signals of MPX infection by interfering with host signalling processes, which results in a decrease in the capacity of cell-mediated immune responses (cytotoxic T cells) to recognize and destroy virus-infected cells. The virotransducer proteins also act intracellularly to inhibit innate antiviral signalling pathways and apoptotic responses to MPX infection. Viromimetics (virokines and viroceptors) are the only type of MPXV proteins that have extracellular roles [134,135]. Both types of viromimicry proteins are involved in regulating antiviral immune responses. Viroreceptors are expressed as cell surface glycoproteins that resemble host immune-related cytokine and chemokine receptors, and bind with them and dysregulate their functions, while virokines mimic host cytokines and chemokines and inhibit their functions [136,137]. MPXV immunomodulatory proteins act synergistically to evade the host antiviral innate immune response through different strategies to allow for viral replication (Figure 3).

Following MPX infection, PRRs such as cytosolic DNA sensors recognize viral DNA and engage defence mechanisms in the host, including production of interferon (IFN) and other pro-inflammatory mediators (IL-6, IL-8, G-CSF) in infected cells and immune cells, and activate the complement cascade [138,139]. Upon MPX infection, the concentration of APCs such as monocytes along with natural killer (NK) cells that directly kill infected cells increases significantly [138,140]. Macrophage-secreted IL-1α and IL-β are elevated in the mild stage of MPX infection, and IL-10, GM-CSF and IL-2R are strongly elevated in the severe stage of infection [141]. Both CD4+ and CD8+ T cells are activated via T cell receptor (TCR) stimulation to recognize APCs and virus-infected cells, respectively. CD8+ T cells are activated on recognition of epitopes derived from F8L protein in MPXV-infected rhesus macaques [142]. In these animals, generation of humoral and cellular immune responses against the virus is characterized by increased levels of B and T cells and production of MPXV-specific IgG and IFN-secreting T cells [143]. These modulations show the immune response to MPX infection, in which pro-inflammatory mediators are secreted to promote migration of immune cells to the site of infection and immunosuppression occurs at the end of aggressive infection through dampened immune responses.

Similarly to other viruses, MPXV has several strategies to evade immune responses. Immunity-related and disease-specific pathways are overexpressed. MPX infection caused by intracellular pathogens or inflammatory processes involves leukocyte chemotaxis or activation of immune cells [84]. MPXV selectively inhibits the expression of genes responsible for cell signalling pathways that activate innate immune responses [144]. IFN is one of the main innate mediators after viral infection, and susceptibility to and severity of the infection are increased when IFN is insufficient [145]. MPXV interferes with IFN signalling pathways through several strategies. It and other poxviruses express a variant form of IFNα/β binding protein, B18, that binds to the cell surface of surrounding uninfected cells and protects cells from the antiviral effects of IFN before cells become infected [146,147]. Studies on VACV have shown that B18 attaches to the cell surface by interaction with glycosaminoglycans, an interaction shared by the IFNα/βBP encoded by MPXV [147,148]. Like VACV, MPXV encodes F3 protein, a homologue of VACV E3 protein that, although truncated, is capable of blocking activation of innate immune cells, thus evading the antiviral IFN system [149]. This truncated E3 protein binds to double-strand RNA (dsRNA) of the virus in infected cells and sequesters it from recognition by PRR, thus inhibiting activation of the protein kinase R (PKR) pathway and supporting viral replication [149]. E3L-specific T cells derived from SPX-vaccinated individuals effectively kill peptide-loaded target cells and VACV-infected cells *in vitro* and the epitopes are shared with MPXV, suggesting E3L as a target protein in vaccine development [150].

In an innate immune response, IL-1β produced by monocytes and macrophages binds to IL-1 receptor and stimulates TNF, IL-2 and other cytokine receptors [151]. A truncated version of BR-209, an IL-1β binding protein, is present in MPXV [49]. BR-209 prevents IL-1β from binding to IL-1 receptors and inhibits the inflammatory cascades. Another host immune defence mechanism, the complement system, is dysregulated by MPXV through genes that encode complement control protein. The MPXV inhibitor of complement enzymes (MOPICE) modulates the antiviral immune response against MPX, as observed by enhanced viral replication *in vivo* and dampened adaptive immune response in a primate model of infection lacking MOPICE expression [139,143]. MOPICE is only expressed in Central African MPXV and is hypothesized as a virulence factor for increased pathogenic properties of this clade compared to the West African clade. Chen et al. [33] compared the sequences of MPXV isolates from West Africa with Congo Basin isolates and identified several possible virulence genes (D10L, D14L, B10R, B14R, B19R) with D14L that encodes MOPICE as the leading candidate. MOPICE inhibits the early steps of the host complement cascade by acting similarly to the mammalian regulators of complement activation (RCA). It mimics the biological activity of complement regulatory proteins that interact with C3b and C4b to inhibit C3 and C5 convertases in the cascades [152]. MPXV also encodes a secreted chemokine binding protein (vCCI), which is abundantly expressed and secreted from MXPV-infected cells. vCCI binds to macrophage inflammatory protein-1 (MIP-1) and inhibits MIP-1-mediated chemotaxis *in-vivo* and *in-vitro* [153].

Although 96% of the MPXV genome is the same as VARV, marked differences in the regions encoding virulence and host range factors have been identified [32]. BR-203, a virulence protein in orthopoxviruses, has a role in avoiding apoptosis of infected lymphocytes. BR-203 is truncated in the West Africa MPXV clade, whereas the full-length gene is found in the Congo Basin MPXV and is speculated to play a role in its higher virulence. Kindrachuk et al. [154] observed that West Africa and Congo Basin MPXV differentially modulate host cell signalling, as portrayed by the differential virulence of the two clades. Congo Basin MPXV selectively downregulates pathways related to apoptosis and cell proliferation, but enhances cell survival compared to the West Africa clade. BR-203 encoding retains MHC-I in the ER and evades the antiviral activity of CD8+ T cells. However, in contrast to interaction with MHC-I, the homologue of BR-203 in MPXV provides immune evasion by inhibiting activation of CD4+ and CD8+ T cells after cognate interaction with infected cells [155]. It is this homologue that is responsible for rendering T cells non-responsive and is identified as MPXV197 [156]. Instead of interfering with antigen presentation or the ability of T cells to respond, MPXV197 directly inhibits T cells through TCR stimulation. Another mechanism is infecting primary human monocytes that are poorly recognized by antiviral CD4+ and CD8+ T cells [155]. However, studies have identified several virulence factors of MPXV that simultaneously regulate host range and immunomodulatory genes in which no individual gene is solely responsible for pathogenicity [157]. Genomic deletion of two particular regions in MPXV effectively inhibits viral replication, tissue spread and mortality *in-vitro* and *in-vivo* with no greater inhibition in either single or dual deletions [157].

## Clinical presentations

**Figure 4.**
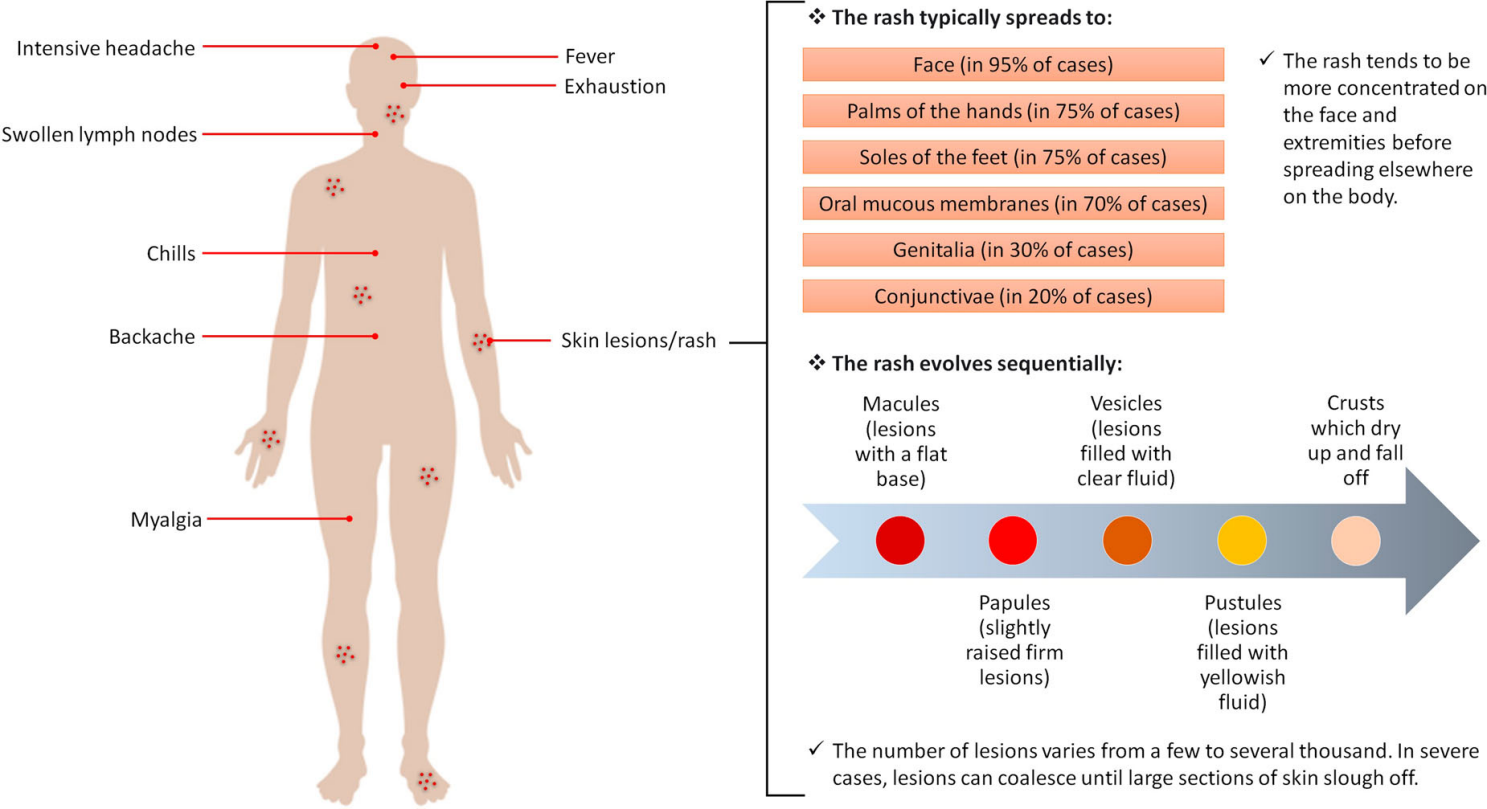
Common symptoms of MPX according to the WHO [160] (Created with BioRender.com).

MPXV incubates for 10–14 days followed by an interval of 1–3 days, during which patients start to suffer from general signs and symptoms of viral infection and the SPX-like skin rash develops [158,159]. MPX disease begins as nonspecific symptoms such as backache, headache, chills, fever, fatigue, myalgia, lethargy and lymph node swelling (Figure 4). After three days, the fever decreases and the rash spreads centrifugally over the body [161,162]. Similarly to SPX rash, it first evolves as macules for 2–4 weeks, and then transforms into papules, vesicles, pustules and finally crusts and scabs [163]. These types of rash can be seen simultaneously during disease progression and last around two to four weeks. The numbers reach up to the thousands, with diameters of 0.5–1 cm and start from the trunk and then spread across the body with a centrifugal pattern of distribution. A centripetal pattern has been reported in a minority of patients [161,162]. Severe lymph node enlargement in the neck, axillary and groin regions are observed and can distinguish MPX from other infections [164]. Onset of rash has been suggested to be the starting point of the infectious period, but the Centers for Disease Control and Prevention (CDC) have stated that this period may start before the appearance of the rash during the prodromal symptoms [165,166].

In severe cases, complications may include conjunctivitis, eye damage resulting from corneal infection, diarrhoea and vomiting resulting in dehydration, encephalitis, tonsillitis, pharyngitis and, uncommonly, bronchopneumonia [161,167,168]. Most reported deaths have been in immunosuppressed patients, young adults and children, with a rate of 1–10%. Some changes have occurred regarding the clinical features and complications after the emergence of vaccines. Higher fatality, more robust rash and more severe clinical presentations have been reported in unvaccinated patients [167,169]. The endemic source also relates to the nature of the disease, as African patients show clinical pictures that are similar but more severe than US cases [162].

Given the nonspecific signs and symptoms of MPX, other diseases should be considered during differential diagnosis, including rickettsia, anthrax, syphilis, measles and scabies, SPX and chickenpox (caused by the varicella-zoster virus (VZV)) [170]. For instance, SPX shows more severe clinical manifestation and evolves as a monomorphic rash (vesicles or pustules), unlike MPX, which emerges polymorphically [171–173]. The lesions of chickenpox are smaller and more superficial than MPX and distributed on the trunk rather than the limbs [170,174]. Lymphadenopathy distinguishes MPX from both SPX and chickenpox.

The virulence of MPXV varies based on the origin of the isolates. The Congo Basin clade has the highest virulence [3,33]. The median lethal dose (LD_50_) of West African MPX is 1.29 × 10^5^, while that of the Congo Basin clade is 5.9 × 10^3^, which was more virulent in the prairie dogs based on morbidity and mortality. Intranasal or intraperitoneal inoculation of adult ground squirrels (*Spermophilus tridecemlineatus*) with 10^5.1^ PFU of West African MPXV leads to anorexia and lethargy within four to five days of infection. Inoculation with Congo Basin MPX is associated with acute severe respiratory tract infection and death within a few weeks. The mortality rate of prairie dogs after inoculation with 10^5.1^ PFU of West African MPXV varies based on the route of viral administration, with a rate of 100% by intranasal and 60% by intraperitoneal inoculation [64,175,176]. Furthermore, in cases infected with Congo Basin clade, MPX caused more frequent skin lesions and cutaneous eruptions [177].

## Transmission modes

Despite the name, MPXV is mainly found in rodents, which are the likely animal reservoir, and this might have contributed to its emergence in humans [178]. However, the natural reservoir of MPXV remains unknown. The human outbreaks in West Africa and elsewhere were transmitted from rodents and other animals due to climate change, rainforest exploitation and highly mobile populations [7]. MPX infection was recognized as a zoonotic disease that infects a wide range of animals, including chimpanzees, lesser and greater white-nosed monkeys, grivets, red colobus monkeys, African brush-tailed porcupines and the Gambian sun squirrel [179,180]. Since MPX is a neglected disease, the pathogenesis in humans is not well studied [181]. MPX is being recognized as an epizootic disease in humans, and is sometimes regarded as a lethal infection [159], with the risk of transmission from human to human [159,182].

**Figure 5.**
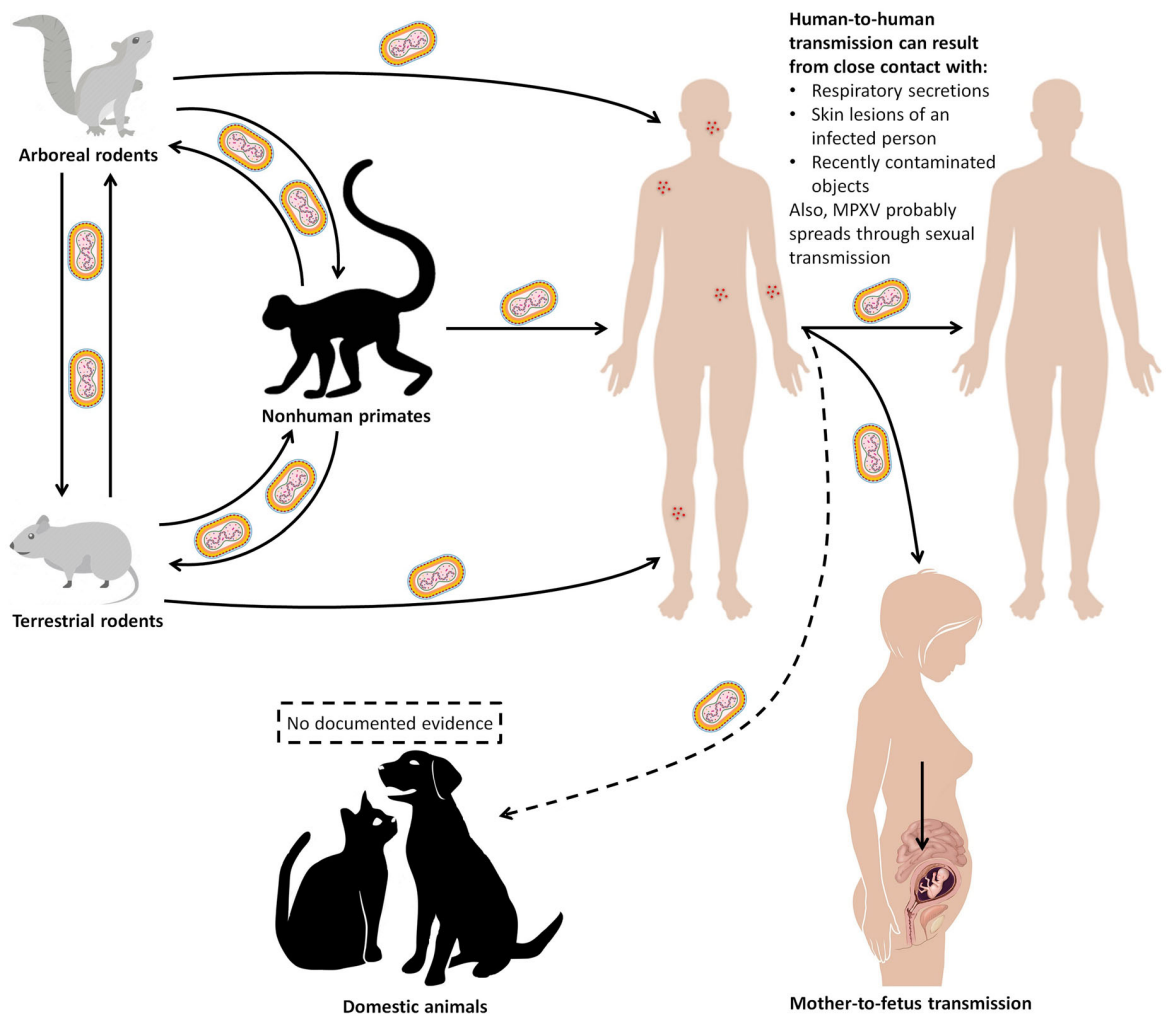
Transmission routes associated with MPXV according to the WHO [160] (Created with BioRender.com).

There are two possible modes of MPXV transmission: human-to-human and animal-to-human (Figure 5). Human-to-human transmission is possible through direct exposure to respiratory droplets and body fluids from infected patients [65,161,162,183–185]. Thus, MPXV can spread through any form of close contact with someone who is infectious, including sexual contact. Also, a pregnant woman can pass the disease to the fetus during pregnancy, or to the newborn after pregnancy by close contact. Studies have shown nosocomial and sexual transmission of MPXV [65,186–188]. Zoonotic infections occur by direct contact with mucocutaneous lesion content, body fluid and blood of infected animals, or even by consuming undercooked meat of an infected animal [65,161,162,183–185].

Human acquired MPXV in most cases is linked with the handling of infected animal tissue [189]; for example, the aetiology of 91% of cases reported in the 1970s (43 of 47 patients) was direct contact with infected animals in comparison with only 9% (4 of 47) following contact with infected humans [29,182]. Reynolds et al. [190] found a significant correlation between the route of MPXV transmission and clinical manifestation. Complex exposure that recognizes groups of patients who were scratched and bitten by infected animals, in addition to exposure to non-invasive virus transmission such as touching or standing close (within 6 feet) to an infected animal or fomite transmission were significantly associated with serious systemic illness and the need for hospitalization, in comparison to non-invasive exposure [190]. Complex exposure is also associated with a lack of febrile prodromes and short incubation periods (9 days in comparison to 13 days in non-invasive exposure) [190].

The MPXV transmission rate, mortality and route of transmission vary based on the virus strain, with the Congo Basin strain having a high transmissibility rate than the West African strain [159]. Congo Basin isolates are more virulent in humans than those isolated from West Africa [33]. Circulating MPXV strains in West Africa and the US have been reported with no fatalities and no human-to-human transmission [191].

## Epidemiology

In the last century, the incidence of human MPX disease was rare and only sporadic cases were reported in several countries in Africa. The first human case of infection with MPX was identified in 1970s in the DRC and linked to a nine-month-old male child [2,192]. This was followed by other sporadic cases reported in 11 other African countries: Cameroon, DRC, Nigeria, Benin, the Republic of the Congo, the Central African Republic, Gabon, South Sudan, Côte d’Ivoire, Sierra Leone and Liberia [160,193]. A large MPX outbreak was reported in the DRC from February 1996 to February 1997 and involved 511 infected cases [194].

In 2003, an MPX outbreak occurred in the US with 47 confirmed or probable cases. It was thought that the infected patients were exposed to the virus through infected prairie dogs kept in a pet distribution facility with other mammals, including the expected first host, African rodents from Ghana [195]. Petersen et al. [161] reported that 116 confirmed cases with a mortality rate of 6.7% and another 280 suspected cases occurred in Nigeria at the end of 2018, with most cases in people below 40.

The incidence of the disease has increased dramatically and the DRC reported a 20-fold increase in the number cases between 1981–1986 (7.2/100,000 population) and 2006–2007 (144.2/100,000 population) and a 5-fold increase from 2001 (0.64/100,000 population) to 2012 (3.11/100,000 population) [196]. Bunge et al. [197] extracted data from 28 peer-reviewed published articles and 15 grey literature reports on human MPX disease and found that the number of cases has increased since the 1970s, with an increase in the median age of infected cases from 4 years old in the 1970s to 21 years old from 2010 to 2019.

In the previous outbreaks, MPX was reported in children and adolescents in the endemic regions and it was believed that the clinical picture and severity of symptoms are the same as those in adults. However, the WHO has recently reported that severe MPX cases occur more commonly among children and are related to the extent of virus exposure. Furthermore, patient health status, nature of complications and underlying immune deficiencies may be associated with worse MPX outcomes [160]. Adults who were born after the 1980s are at increased risk because vaccination against SPX, which may protect against MPX, ceased after eradication of SPX in the 1980s [7]. Moreover, it was believed that MPX infects males and females equally, but many cases of MPX have occurred in men who have sex with other men (MSM) in the current multi-country outbreak. The CDC reported that MSM make up the majority of MPX cases in the 2022 outbreak, which puts gay, bisexual and transgender people at increased risk of MPX infection [166]. Uncertainty remains on the sexual transmission routes of MPXV between MSM and further studies are needed to better understand this risk.

Coinfection of MPX with other sexually transmitted diseases (STD) and blood borne pathogens has been reported [198], but patients with human immunodeficiency virus (HIV) have the most concern since infection with HIV is considered to be a risk factor for MPX during the current outbreak [199,200]. Lack of appropriate immune response in cases of advanced or uncontrolled HIV infection is significantly associated with a poor prognosis, longer duration of MPX signs, delayed curing of self-limiting MPX infection, other comorbidities, and complicated treatments [198,201]. Therefore, screening of MPX patients for HIV is highly recommended in MSM [198]. Infection with MPX has also recently been recognized as a factor that increases the probability of HIV infection [202–204]. A recent cross-sectional descriptive study from Madrid, Spain found that 44.3% (225/508) of confirmed MPX cases also had HIV infection [201]. Another study from London, UK showed that 35.9% (70/195) of confirmed MPX cases had concomitant HIV infection [205]. Also, mild MPX infections among HIV/AIDS patients have been reported from Portugal and Italy [206,207], especially among individuals with increased T-helper cells count, undetectable HIV viral genetic material, and under anti-retroviral therapy [208]. Patients with immunosuppression caused by HIV had a distinct wide spectrum of clinical manifestations concurrently with typical MPX lesions. Exanthema, fever, genital ulcers and inguinal lymphadenopathy were significant in MPX patients during the ongoing outbreak in Portugal [206]. Papules, pustules, umbilicated with a necrotic central lesion in the perianal area, genitals, mouth, trunk and face were reported in a 24-year-old bisexual man with acute HIV infection [209]. Furthermore, during the 2017–2018 MPX outbreak in Nigeria, more than half of MPX deaths were in patients with uncontrolled HIV with AIDS manifestations who were not receiving antiretroviral therapies [210]. Another study from Nigeria found that HIV-coinfected MPX cases had more prolonged illness, larger lesions, and higher rates of both secondary bacterial skin infections and genital ulcers, compared to HIV-negative MPX cases [211]. Coinfection with another STD was also reported among HIV/MPX patients. A patient with undiagnosed advanced HIV was recently reported with syphilis, and presented with nasal necrosis, severe penis and oral mucosa infections, and MPX lesions distributed over the whole body [212].

Active MPX disease surveillance was conducted in nine health zones in central DRC from November 2005 to November 2007 and 760 laboratory-confirmed cases of MPX were found, with an overall annual incidence of 55.3/100,000 population. Male gender, age <15, no SPX vaccination and living in forested areas were major risk factors for infection [213]. In 2017, a large outbreak of MPX was reported in Nigeria, with over 500 suspected, over 200 confirmed cases, and a mortality rate of 3% [214]. In another study, Beer and Rao [215] analyzed 71 documents describing MPX cases and outbreaks between 1970 and 2018. The reported outbreaks were found to have increased since 1970, with a total of 35 reported outbreaks outside the DRC, including 20 between 2010 and 2018. Table 3 provides data on human MPX cases and deaths by country from previous outbreaks.

**Table 3.** Number of MPX cases and deaths from 1970 to 2018.

Country	Time frame	Total suspected cases	Total deaths	References
Democratic Republic of the Congo	1970	1	1	[1]
	1981–1986	338	33	[179]
	1996–1997	773	8	[216]
	2001	388	13	[196]
	2002	881	14	[196]
	2003	755	16	[196]
	2004	1024	29	[196]
	2005	1708	26	[196]
	2006	783	20	[196]
	2007	970	11	[196]
	2008	1599	67	[196]
	2009	1919	27	[196]
	2010	2322	26	[196]
	2011	2208	15	[196]
	2012	2629	34	[196]
	2013	2460	37	[196]
	2016	155	11	[217]
	2019	3794	73	[218]
	2020	4594	171	[218]
Central African Republic	2001	8	2	[219,220]
	2010	2	0	[219]
	2015	3	1	[220]
	2015–2016	62	5	[221,222]
	2017	8	0	[193]
	2018	33	1	[223]
Republic of the Congo	2003	12	1	[224]
	2010	11	1	[225]
	2017	88	6	[193]
Sudan	2005	37	0	[226]
Cameroon	1989	1	0	[227]
	2018	16	0	[223]
Gabon	1987	1	1	[228]
	1991	9	0	[193]
Nigeria	1971	2	0	[177]
	1978	1	0	[177]
	2017–2018	228	6	[65]
Sierra Leone	1970–1971	1	0	[177]
	2014	1	1	[193]
	2017	1	0	[193]
Liberia	1970–1971	4	0	[177]
Côte d’Ivoire	1971	1	0	[229]
USA	2003	47	0	[230,231]
	2021	2	0	[232]
UK	2018	4	0	[233]
	2019	1	0	[234]
	2021	3	0	[235]
Singapore	2019	1	0	[236]

Source: Adopted from Brown and Leggat [162]; Beer and Rao [215]; Adegboye et al. [162,215,237].

On 5 August 2022, the CDC reported 28,220 confirmed cases in 88 countries since 1 January 2022 [238]. Most of these cases (27,875) were reported from 81 countries that have not historically reported MPX [238]. Furthermore, one month ago, the WHO reported several outbreaks of human MPX in regions including the Americas, the Eastern Mediterranean, Europe and the Western Pacific, with a total of 1285 laboratory-confirmed MPX cases. while 1536 suspected and 59 confirmed MPX cases with 72 deaths occurred in Africa from January to June 2022 [232]. Table 4 provides data on global MPX cases and deaths by country from the multi-country 2022 outbreak. Several ecological and environmental factors may have contributed to the emergence or re-emergence of MPX infection, including exploitation of rain forests, climate change, geopolitical and armed conflicts in disease regions, waning herd immunity, highly mobile populations, and the end of SPX vaccination [7,239].

**Table 4.** MPX cases and deaths reported by the WHO during the multi-country 2022 outbreak (as of 8 June 2022) [232].

WHO Region	Country	Confirmed cases	Suspected cases	Deaths
AFRO	Cameroon	3	28	2
	Central African Republic	8	17	2
	Republic of Congo	2	7	3
	DRC	10	1356	64
	Liberia	0	4	0
	Sierra Leone	0	2	0
	Nigeria	31	110	1
	Ghana	5	12	0
AMRO	Argentina	2	0	0
	Canada	110	0	0
	Mexico	1	0	0
	United States of America	40	0	0
EMRO	United Arab Emirates	13	0	0
	Morocco	1	0	0
EURO	Austria	1	0	0
	Belgium	24	0	0
	Czech Republic	6	0	0
	Denmark	3	0	0
	Finland	3	0	0
	France	66	0	0
	Germany	113	0	0
	Hungary	2	0	0
	Ireland	9	0	0
	Italy	29	0	0
	Israel	2	0	0
	Latvia	2	0	0
	Malta	1	0	0
	Netherlands	54	0	0
	Norway	2	0	0
	Portugal	191	0	0
	Slovenia	6	0	0
	Spain	259	0	0
	Sweden	6	0	0
	Switzerland	12	0	0
	The United Kingdom	321	0	0
WPRO	Australia	6	1	0
Cumulative	36 countries	1344	1537	72

AFRO, Africa; AMRO, Americas; EMRO, Eastern Mediterranean, EURO, Europe; WPRO, Western Pacific; The DRC, Democratic Republic of the Congo

The MPX epidemic threshold is <1, representing the average number of cases caused by an infected person. Thus, MPX infection is likely to be limited to small outbreaks, instead of affecting the whole population. However, the worldwide decline of general orthopoxviral immunity has increased susceptibility to MPX infection [189,213]. In addition, there might be new genetic mutations, leading to widescale outbreaks of MPX [240]. Trend monitoring in 2011–2012 showed that the epidemic threshold of MPX had increased to 1.25 new cases, posing a high risk for health security. Yet, the exact transmission period of MPX, in which it might have been spreading for months or years, remains unknown. MPX has occasionally been endemic in the West and Central Africa areas, with a high number of cases being recently reported. This virus and other orthopoxviruses were commonly controlled through a combined containment strategy [241], and there is reliance on previous and current findings to contain the outbreak.

## Laboratory diagnosis

Rapid diagnosis is crucial to eradicating an outbreak, but clinical manifestations are not accurate enough to give a definitive diagnosis. In MPXV-endemic areas with limited resources, a serological test for MPXV-specific antibodies was used before real-time PCR became available [242]. Therefore, the need for diagnostic tools has appeared. Specimens should be taken from skin exudate, vesicular lesions, or crusts and kept cold in a sterile and dry tube. To date, detection of MPXV DNA from extracted nucleic acid using real-time PCR assays is the preferred laboratory method due to its high sensitivity and accuracy [160]. Diagnosis can be confirmed by virus isolation from nasopharynx and oropharynx secretions [161]. Skin biopsies can be obtained from the intact lesion roof or vesiculopustular rash. Certain sera are required in serologic tests to detect the specific immunoglobulin M and G (IgM and IgG) of MPX within 5 and 8 days, respectively [161]. Although this type of testing gives evidence of viral exposure, it also reveals an immune response following vaccination or exposure to other orthopoxviruses [243]. Developing new techniques with more immunological sensitivity could enhance the diagnosis. Some diagnostic tools require large and well-prepared laboratories, but many countries, especially those with the main burdens of the disease, cannot offer these facilities. Accordingly, point-of-care tests are needed without high levels of training suitable for basic laboratories.

Immunohistochemistry (IHC) and histology of common lesions reveal acanthosis, dermal perivascular infiltration, basal vacuolization and keratinocyte necrosis. Spongiosis, ballooning degeneration, epidermal necrosis, viral inclusion, giant cells with neutrophils and eosinophils, and signs of vasculitis are also seen in vesicular lesions. Electron microscopy shows intracytoplasmic structures that are sausage-shaped and oval-to-round inclusions [161].

Haematoxylin and eosin (H & E) stains are used to examine formalin-fixed, paraffin-embedded skin biopsy specimens of MPX infection [244]. Human MPX is histologically characterized by ballooning degeneration of basal keratinocytes and a mild acanthotic spongiotic epidermis that develops into full-thickness skin necrosis of a markedly acanthotic epidermis, containing several viable keratinocytes [245]. The epidermis and superficial dermis are composed of moderate inflammatory infiltrate cells (lymphocytes and neutrophils) with the presence of large multinucleated cells and rare eosinophilic viral inclusion bodies [246]. The keratinocytes exhibit multinucleation with nuclear moulding due to chromatin margination in the epidermis region [230]. In one case report, the papulonecrotic stage of MPX showed early evidence of vesiculation with minimal pustulation. Cell necrosis destroyed the stratum basale layer, while marked hyperplasia and intracellular oedema of stratum spinosum aggregated the papule, leading to formation of spindle cells [244,247]. The rete ridges surrounding the dermal papule were four times deeper with doubled cell layers and an extended area of affected stratum spinosum, in comparison to normal skin. Shallow incomplete stratum granulosum development under the stratum corneum has also been observed [247].

**Figure 6.**
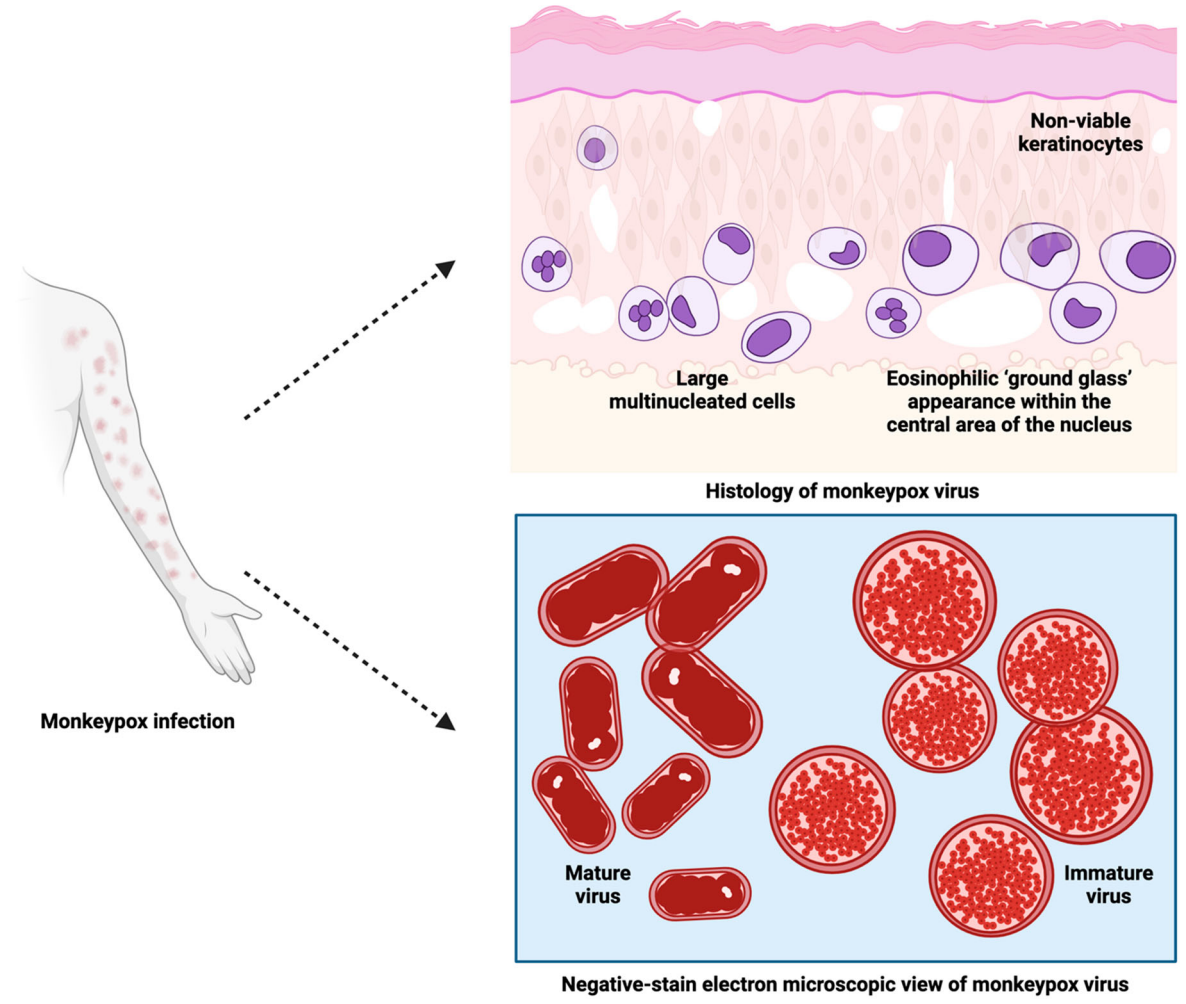
Histology and negative-stain electron microscopic views of MPXV virions (Created with BioRender.com).

In a novel respiratory model of infection with MPX, the histologic manifestation of the progressing inflammatory lung was correlated with the dose of administered virus. The animal model which survived longest after MPX infection showed distinctive necrotic areas with multifocally fibrin-filled alveoli in the lung, pulmonary fibrosis and oedema, tracheal congestion and fibrous pleural adhesions [248]. The orthopox viral antigen has been detected in degenerating keratinocytes and follicular epithelium of skin biopsy specimens by rabbit anti-VACV polyclonal antibody IHC staining [245]. These findings are supported by the presence of spherical Guarneri intracytoplasmic inclusion bodies located at the affected keratinocytes and their absence in the uninvolved epidermis at the edge of the bullae [230]. A dual IHC staining study of the virus in two animal models showed the presence of abundant viral antigens in most organs and highlighted the colocalization of apoptosis with poxvirus antigen [249]. Both immature and mature stages of assembled virions within the cytoplasm of keratinocytes of glutaraldehyde-fixed skin biopsy human specimens have been observed under transmission electron microscopy. The cross-sections of mature virions have dumbbell-shaped features, and brick-shaped virions with regularly spaced, threadlike ridges on the exposed surfaces have been viewed on negative-stain electron microscopy (Figure 6) [250]. In general, the lesions of MPX are identical to other viral exanthems such as cowpox virus (CPXV), VARV, VZV, tanapox and herpes simplex virus (HSV) [251].

## Current treatment and prevention protocols

During the MPX epidemic in the US in 2003, the CDC stated that taking an SPX vaccine up to two weeks after MPX exposure could reduce the symptoms but not prevent disease [252]. However, the SPX vaccine is neither available to the public nor given to infected patients. This is attributed to concerns over giving a live VACV, its cost and the unknown adverse events among immunocompromised patients [187,252,253]. Patients with low immunity are at high risk of serious side effects from vaccination, including cryptococcal meningitis, cardio-related complications, pneumonia and progressive VACV, which is a rare side effect leading to tissue and skin destruction and can be fatal [254–257]. Second and third generations of SPX vaccines ACAM2000 and Imvamune have been developed, [258] but ACAM2000 has cardiac side effects similar to those reported with the first-generation vaccine [259] and there is no information about its safety among HIV patients [256]. In 2015, the CDC stated that HIV patients and those with a CD4 cell count of 50–199 cells/mm^3^ who were exposed to SPX should take Imvamune, while ACAM2000 should be given to those with a CD4 cell count of >200 cells/mm^3^. Animal studies have shown that ACAM2000 offers higher viral suppression than Imvamune [256].

There is no specific approved treatment for MPX. Management is limited to treating secondary bacterial infections, reducing the symptoms and giving supportive care [161]. However, there are two drugs, CMX001 and ST-246, developed for the treatment of SPX. ST-246 (tecovirimat) has been approved by the FDA for SPX and has shown efficacy against MPX [260]. Berhanu et al. [261] reported that ACAM2000 alone after MPX exposure was less effective than ST-246 alone or combined with ACAM2000. In a study of the overlapping effects of ACAM2000 and ST-246, the efficacy of the vaccine was largely unaffected, but the humoral response was reduced. Thus, ST-246 should not be given concurrently with ACAM2000 [262]. CMX001 (Brincidofovir) has shown promising results in animal models with various poxviruses including MPX [260]. According to the ClinicalTrials.gov database, as of 19 June 2022, there are four registered trials to evaluate the efficacy of anti-MPX agents (Table 5).

**Table 5.** List of clinical trials of drugs and vaccines against MPX.

Product	Identifier	Type	Developer, Country	Trial location	Status	First Posted
IMVAMUNE®	NCT02977715	SPX vaccine (attenuated live virus)	Bavarian Nordic, Denmark	DRC	Active, not recruiting	30 November 2016
Imvamex®	NCT03745131	SPX vaccine (attenuated live virus)	Bavarian Nordic, Denmark	United Kingdom	Completed	19 November 2018
ST-246®	NCT00728689	Anti-orthopoxvirus compound inhibits release of extracellular virus by targeting the F13L W protein	SIGA Technologies, Inc., United States	United States	Completed	6 August 2008
Tecovirimat (TPOXX, previously ST-246)	NCT02080767	Anti-orthopoxvirus drug that interferes with a p37 viral protein	SIGA Technologies, Inc., United States	-	Available (FDA-approved)	6 March 2014

Data were collected from https://clinicaltrials.gov/.

ST-246®, 4-trifluoromethyl-N-(3,3a,4,4a,5,5a,6,6a-octahydro-1,3-dioxo-4,6-ethenocycloprop [f]isoindol-2(1 H)-yl)-benzamide; DRC, Democratic Republic of the Congo.

According to the CDC, the following actions help prevent the spread of MPX [166]: avoiding direct contact with individuals who have a rash and flu-like symptoms and appear to be MPX-infected; and avoiding sharing objects and materials (e.g. toilet seats, doorknobs, dishes, bedding, towels or clothing) with someone who has MPX. Also, it is highly recommended to wash hands often with soap and water or use a sanitizer containing alcohol for hand rubbing, especially after using the bathroom or being outside, and before touching the eyes, nose, mouth or face, and before preparing or eating food. Furthermore, people should avoid contact with animals that can get MPX, especially primates and rodents, and even touching their bedding materials. People with probable or confirmed MPX should avoid contact with animals, including pets, to prevent the spread of MPX.

### Potential therapeutics

Oral inhibitors of orthopoxvirus infections such as ether lipid prodrugs of cidofovir (CDV) and (S)-HPMPA, ST-246, N-meth-anocarbathymidine (N-MCT) and SRI 21950 (a 4′-thio derivative of iododeoxyuridine) have potentially beneficial effects. HPMPO-DAPy is another high-activity compound that requires parenteral delivery [263].

At doses within a pharmacologically feasible range, CDV, cyclic HPMPC (cHPMPC), HPMPA, ribavirin, tiazofurin, carbocyclic 3-deazaadenosine, 3-deazaneplanocin A and DFBA (1-(2,4-difluorobenzyloxy)adenosine perchlorate), a derivative of adenosine N1-oxide, all inhibited replication of all three VARV strains and the other orthopoxviruses. Two other compounds – methisazone and bis-POM-PMEA – had a weaker antiviral effect. Studies on the sensitivity of 35 strains of VARV and other orthopoxviruses to a subset of three of the most active compounds – CDV, cHPMPC and ribavirin – to examine possible natural drug resistance among VARV isolates obtained from different geographical regions and at different times suggest that nearly all isolates have similar sensitivity [264].

Since 1996, CDV ((S)-1-(3-hydroxy-2-phosphonylmethoxypropyl) cytosine, HPMPC) has been approved for clinical use in AIDS patients with cytomegalovirus (CMV) retinitis. CDV is particularly effective against all DNA viruses. VACV, VARV, CPXV, MPX, camelpox (CMPV), molluscum contagiosum and orf (sheep pox) are among the poxviruses [265] that are sensitive to the inhibitory effects of CDV. These findings suggest that CDV may be useful in treatment and short-term prevention of SPX and kindred poxvirus infections [266–268].

Starting antiviral treatment 24 h after lethal intratracheal MPX infection using either the antiviral agent (CDV) or a related acyclic nucleoside phosphonate analogue (HPMPO-DAPy) and different systemic treatment regimens leads to lower mortality and reduced cutaneous MPX lesions. In contrast, no significant reduction in mortality was seen when monkeys were vaccinated 24 h after MPX infection with a standard human dose of a currently recommended SPX vaccine (Elstree-RIVM). All surviving animals had virus-specific blood antibodies and antiviral T cells after antiviral treatment was stopped 13 days after infection. These findings suggest that effective biological threat preparedness should include the ability to treat exposed people with antiviral substances such as CDV or other selective anti-poxvirus medications [269].

The question of how to apply this knowledge to human poxvirus infections remains unresolved. Findings show that the currently recommended CDV dose of 5 mg/kg body weight per week in combination with probenecid (which reduces nephrotoxicity) is unlikely to protect people who have been exposed to VARV infection. It was further shown that the antiviral action is unaffected by the drug delivery schedule. To have a robust protective effect, CDV exposure must be 5–10 times higher than that currently given to patients. However, such high doses may cause nephrotoxicity, and regiments with probenecid administration and dosing schedules that may alleviate CDV uptake into renal tubular epithelial cells should be considered [270].

When given as early as 5 days before infection or as late as 3 days after with CPXV or VACV (as orthopoxvirus examples), a single dose pre- or post-treatment of mouse models with CDV at 3–100 mg/kg proved successful. Interval treatments with 6.7 or 2 mg of CDV/kg given every third day beginning 72 h after infection were also effective. All mice infected intraperitoneally with ectromelia virus (EV, mousepox) and treated for 7–30 days with CDV died during or after treatment; however, most treated groups experienced significant delays in time to death, and reduced virus proliferation in organs and no CDV resistance was identified [271].

The efficacy of CMX001 (an analogue of CDV; Table 6) as a preventive and early illness antiviral has been tested using rabbitpox virus (RPV) infection of New Zealand white rabbits as a model for SPX. The findings should also apply to MPX infections and the treatment of SPX vaccination side effects [278].

**Table 6.** Promising anti-MPX medications.

Name	Structure and nature	Evidence	Other Uses and notes	Combined therapy	Side effects
Cidofovir, also called HPMPC or Vistide [269]	[(S)-1-(3-hydroxy-2-phosphonylmethoxypropyl)cytosine, HPMPC] [266] acyclic cytosine phosphonate analogue [272]	Cidofovir has been successfully used in humans to treat persistent molluscum contagiosum and orf in immunocompromised individuals using topical and intravenous routes [266] Targets the viral DNA polymerase and inhibits poxvirus replication *in-vitro*, *ex-vivo* and *in vivo* [272] Effective against almost all DNA viruses and has a broad spectrum of activities [266–268] Starting antiviral treatment 24 h after deadly intratracheal MPX infection using either of the antiviral drugs and a variety of systemic treatment regimens resulted in significantly lower mortality and cutaneous MPX lesions. All surviving animals had virus-specific blood antibodies and antiviral T cells after the antiviral medication was stopped 13 days after infection [269] Three non-human primates were pox-antigen negative after exposure to a fatal dose of MPX and receiving cidofovir. All three primates survived [273]	Resistant orthopox viruses were thought to be untreatable with cidofovir; however, their virulence might be reduced [274] When given as early as 5 days before infection or as late as 3 days after infection with either CPXV or VACV, a single dose pre- or post-treatment with cidofovir at 3–100 mg/kg in mouse models proved successful [271]	Coadministration of cidofovir and Dryvax in a single dosage efficiently minimized vaccination side effects against MPX but severely compromised vaccine-elicited immunological responses and vaccine-induced immunity [275]	Exposure to cidofovir that will have a protective effect against certain orthopox viruses (i.e. VARV major virus) will need doses that are 5–10 times that currently administered to humans, such doses may lead to nephrotoxicity [267]
HPMPA	((S)-9-(3-hydroxy-2-phosphonylmethoxypropyl)adenine) [264]	HPMPA was effective against several orthopoxviruses, with IC50s of 10.9 and 9.2 μg/ml for MPX Vero and LLC-MK2 infected cells, respectively [264] *In vitro*, (S)-HPMPA is active against numerous orthopoxviruses, but not *in vivo*. Ether lipid esters, such as ODE-(S)-HPMPA and HDP-(S)-HPMPA, exhibit better bioavailability and activity against CPXV and VACV infections in mice [276]	HPMPA is closely linked to cidofovir and works similarly to prevent viral replication. Because cidofovir has already been approved for use in the US and is far more likely to be used to treat poxvirus infections, HPMPA is unlikely to be used in US clinical trials [264]		Oral HDP-(S)-HPMPA and ODE-(S)-HPMPA showed no effect. ODE-(S)-HPMPA, given 72 h after CPXV infection or 24 or 48 h after VACV infection, also reduced mortality. Both compounds have the potential to cure human cases of SPX vaccination-related adverse effects [276]
Brincidofovir (CMX001) HDP-cidofovir	Lipophilic nucleotide analogue formed by covalently linking 3-(hexdecyloxy)propan-1-ol to cidofovir (hexadecyloxypropyl ester of cidofovir) [277]	In comparison to CDV, CMX001 has significantly improved efficacy against all dsDNA viruses [278] Antiviral treatment with CMX001 and ST-246 protects mice infected with the murine MPX in a STAT1-deficient C57BL/6 mouse model [279] CMX001 is effective for symptomatic RPV infection (an orthopoxvirus infection) [280]	Orally available [278]	When administered together on the day of infection, CMX001 and ST-246 provide protection [279]	To date, there has been no evidence of nephrotoxicity in healthy volunteers or critically ill transplant patients [278]
Tecovirimat (ST-246) [281]		Tecovirimat is a new antiviral that targets viral p37 protein orthologs to prevent orthopoxviruses from egressing [281] Has antiviral activity in a variety of animal models [272]	Tecovirimat works as an antiviral by suppressing the generation of EVs, which prevents cell-cell and long-distance propagation [282]	ACAM2000 (SPX vaccine) given after exposure did not protect against severe MPX disease or death, while post-exposure treatment with tecovirimat alone or with ACAM2000 gave complete protection. Tecovirimat after infection was 83% (days 4 and 5) or 50% (day 6) effective [261]	Following a phase I clinical investigation, safety was validated in healthy human volunteers [272]
Retro-2 and analogues		PA104 was the most effective Retro-2 analogue, suppressing viral propagation by 90% at 1.3 M with a good selectivity index. These findings, and additional identification of PA104’s specific protein targets and *in-vivo* activity, could be significant for development of effective antivirals for OPXV [282]			PA104 inhibited two different ST-246-resistant viruses, indicating that it could be useful in combination therapy with ST-246 [282]
siRNAs		At a dose of 1 nM, siRNAs siB1R-2 and siG7L-1 reduced MPXV viral proliferation by 95%. Without inducing a beta interferon response, siB1R-2 and siG7L-1 silenced their corresponding transcript: B1R and G7L mRNAs [283] Seven siRNA constructs suppressed viral replication in cell culture by 65-95% with no apparent cytotoxicity, targeting either an essential gene for viral replication (A6R) or a key gene in viral entry (E8L). Further tests using wild-type and recombinant MPX producing green fluorescent protein revealed that siA6-a was the most powerful construct, inhibiting viral replication for up to 7 days at a dose of 10 nM [284] Different pathogenic orthopoxviruses (CPXV and MPXV) were inhibited up to 70% at the lowest concentration (1 nM) tested, indicating siD5R-2 (siRNA targeting D5 protein) efficacy. siD5R-2 had antiviral effects in human keratinocyte and fibroblast cell cultures infected with VACV [285]		When siB1R-2, siG7L-1, or siD5R-2 were coupled with cidofovir, strong synergistic effects were seen. Combination therapy of siRNA and cidofovir could be effective in treating poxvirus infections [283]	
Mycophenolic acid (MPA)		In plaque reduction experiments, MPA suppressed CMPV, CPXV, MPXV and VACV by 50% in African green monkey kidney (Vero 76) and mouse 3T3 cells at 0.2–3 μM [286]	Anti-orthopoxvirus efficacy of ribavirin is boosted by other modes of virus inhibition. Biological variations in mode of action and the immunosuppressive potential of ribavirin and MPA, cause the former to be effective against orthopoxvirus infections in animals, while the latter is not [286]		MPA and ribavirin were more toxic to replicating cells than stationary cell monolayers, with greater toxicity in 3T3 cells than Vero 76 cells. Compared to Vero 76 cells, the higher antiviral efficacy and toxicity of ribavirin were due to more accumulation of mono-, di- and triphosphate forms of the drug in 3T3 cells. Virus inhibition was linked to suppression of intracellular guanosine triphosphate pools for MPA and ribavirin [286]
Ribavirin		Ribavirin was significantly more effective against these viruses in 3T3 cells (IC50 2–12 μM) than in Vero 76 cells (IC50 30–250 μM) [286]			

The efficacy of a single dose of CMX001 at 20, 25 or 30 mg/kg doses administered on days 4, 5, 6 and 7 post-infection was examined in A/Ncr mice intranasally infected with modest doses of EV (<20 PFU). To track disease progression, the mice were evaluated for weight loss, blood interferon levels, alanine aminotransferase (ALT), aspartate aminotransferase (AST), viral DNA copies and neutrophilia levels. It was discovered that a single dosage of 25 mg/kg of CMX001 given on days 4 or 5 after infection was effective in curing deadly mousepox [277].

ST-246, developed by SIGA Technologies Inc. under licence from ViroPharma Inc., has been found to disrupt a vital phase in morphogenesis of orthopoxviruses. The antiviral activity of ST-246 has been proven in a variety of animal models and its safety was confirmed in healthy human volunteers in a phase I clinical trial [272].

Small compounds like Retro-2 have been shown to reduce orthopox infection *in vitro* and to a lesser extent *in vivo* by inhibiting the retrograde pathway. A vast panel of drugs with a benzodiazepine scaffold-like Retro-1 have been screened to find more effective retrograde pathway inhibitors. When compared to Retro-1, a subset of these compounds had superior anti-VACV activity, resulting in a reduction in extracellular virus (EV) particle formation and viral dissemination [282].

Mycophenolic acid (MPA) and ribavirin, two inhibitors of cellular inosine monophosphate dehydrogenase, have been tested for inhibitory efficacy against orthopoxviruses. 6-aziridine, CDV (HPMPC) and cyclic HPMPC were among the unrelated anti-poxvirus drugs studied for comparison. In plaque reduction experiments, MPA suppressed CMPV, CPXV, MPX and VACV by 50% in African green monkey kidney (Vero 76) and mouse 3T3 cells at 0.2–3 μM. Ribavirin was significantly more effective against these viruses in 3T3 cells (50% inhibition at 2–12 μM) than in Vero 76 cells (inhibitory at 30–250 μM) [286]. Table 6 lists the most promising anti-MPX medications, along with evidence for their efficacy, uses, adverse effects, and use in combination therapy.

### Vaccines

SPX eradication, managed by the WHO and certified 40 years ago, resulted in most countries discontinuing routine SPX vaccination. Over 70% of the world’s population is now thought to be unprotected against SPX and related orthopoxviruses like MPX [7].

In 2018, an outbreak occurred in the UK, but there was little motivation to introduce SPX vaccines to provide cross-protection against MPX [287]. In June 2019, an ad hoc and unofficial group of interested specialists gathered at Chatham House in London to discuss these problems, reviewing available data and identifying MPX-related research needs. It was agreed that a better understanding of the genomic evolution and changing epidemiology of orthopoxviruses, the utility of in-field genomic diagnostics and the best disease control strategies such as vaccination with new generation non-replicating SPX vaccines and treatment with recently developed antivirals were all necessary [7]. Anti-orthopoxvirus IgM and alterations in anti-orthopoxvirus IgG, CD4, CD8, or B-cell responses were found in previously vaccinated MPX cases as indications of a new infection. In MPX cases (vaccinated and unvaccinated), anti-orthopoxvirus IgM and CD8 responses were the most common, with IgG, CD4 and memory B-cell responses indicating vaccine-derived immunity. Immune markers revealed the presence of asymptomatic illnesses in both vaccinated and uninfected people [288].

Active population-based surveillance has been carried out in nine health zones across the DRC. Vaccinated people had a 5.2-fold decreased risk of MPX than those who had not been vaccinated. A comparison of active surveillance data from the 1980s and 2006–2007 in the same health zone showed a 20-fold rise in human MPX incidence. This incidence has risen rapidly in rural DRC 30 years after mass SPX vaccination campaigns ended [213].

Human MPX outbreaks in Africa and the 2003 outbreak in the US have demonstrated that naturally occurring zoonotic orthopoxvirus illnesses remain a public health problem. Vaccination could minimize much of the hazard provided by orthopoxviruses, but because the SPX vaccine is a live orthopoxvirus vaccine, the vaccine can pose a major health risk [289]. Due to a high degree of sequence conservation, vaccinating with VACV also prevents MPX. Antigens within the MPX proteome that contribute to immune responses have yet to be identified in detail [290]. In the past, people who had been exposed to SPX were treated with the SPX vaccine and VACV immune globulin (VIG). Patients who were at high risk of problems following SPX immunization were also given VIG. As a result, post-exposure vaccination and VIG therapies may become again essential therapeutic options [291].

A protein microarray was used to capture antibody responses to MPX infection and human SPX vaccination. Only 14 of these proteins were recognized by IgG from vaccinated humans, but serum IgG from cynomolgus macaques recovering from MPX recognized at least 23 proteins within the orthopox proteome. Twelve of the 14 antigens discovered by human vaccines were also recognized by convalescent macaque IgG. The structural proteins F13L and A33R and the membrane scaffold protein D13L had the highest level of IgG binding. Before onset of clinical symptoms, significant IgM responses to the MPXV’s A44R, F13L and A33R were observed. Antibodies from vaccination recognized a limited number of proteins shared with pathogenic virus strains, although humoral responses to antigens specific to the MPXV proteome were also required for recovery from infection [290]. Different types of vaccines and their potential uses in prevention and treatment of MPX are shown in Table 7.

**Table 7.** Vaccines of various sorts and their potential applications in the prevention and treatment of MPX.

Vaccine	Efficacy against MPX	Notes
First-generation SPX vaccines: Dryvax and live VACV strains like Lister, Copenhagen (Cop), chorioallantois VACV Ankara (CVA), Tian Tan (TT), Bern and New York City Board of Health (NYCBH)	– Dryvax (freeze-dried vaccine) protects against SPX and MPX [292] – Due to a high degree of sequence conservation, vaccinating against SPX with VACV may protects against MPX [290]	– Associated with serious complications in both naïve and immune people [293]. First-generation SPX vaccines were used in the eradication program. These VACV strains caused varying levels of vaccine-related complications and as a result, strains like Bern and Copenhagen were favoured less than Lister and NYCBH [294] – Coadministration of cidofovir and Dryvax in a single dose greatly reduced adverse effects, but severely compromised vaccine-elicited immunological responses and vaccine-induced immunity to MPX [275] – Contraindicated in immunocompromised people [292]
VACV vaccine strain LC16m8	– Although lesions were smaller than those induced by the original Lister strain, LC16m8 is less attenuated than MVA and retains the potential to multiply and cause lesions in human vaccinees [295] – Due to a frameshift mutation, LC16m8 does not express B5R protein [296]. As a result, a tiny plaque phenotype develops. Humans immunized with LC16m8 have inadequate immune responses to this protein [297], which is a key target for antibodies that neutralize the EEV form of VACV [298] – Efficacy was compared to that of the original Lister strain. Monkeys were immunized with LC16m8 or Lister and subsequently infected with MPXV strain Liberia or Zr-599 intranasally or subcutaneously. With intranasal-inoculation, immunized monkeys had no symptoms of MPX, but nonimmunized controls showed normal symptoms. With subcutaneous injection, monkeys immunized with LC16m8 showed no signs of MPX except for a small ulcer at the site of MPXV inoculation, while nonimmunized controls showed fatal and typical symptoms. These findings imply that LC16m8 protects monkeys from deadly MPX and may elicit protective immunity against SPX [299]	– Derived from Lister strain in Japan [294] and harbours a mutation in the critical membrane protein B5R [299]
Modified VACV Ankara (MVA); also known as Imvanex in the European Union, Imvamune in Canada, and Jynneos in the United States [300]	– In a STAT1-deficient C57BL/6 mouse model of MPX, vaccination with MVA followed by a booster vaccine protect against an intranasal MPX challenge and elicits a more robust immune response than a single vaccination [279] – In humans and immunocompromised animals, it is safe. The MVA-based SPX vaccine protected macaques against a deadly respiratory challenge of MPX, making it a promising contender for human protection [293] – To produce immune responses comparable to those induced by the initial SPX vaccines, higher viral titres and multiple doses are required [301–303] – Before MPX viral challenge, there was no significant difference in neutralizing antibody levels in animals vaccinated with a single ACAM2000 immunization (132 U/ml) vs. a prime-boost Imvamune regimen (69 U/ml) [304]	– A highly attenuated replication-deficient strain of VV [293] derived from strain CVA by Mayr and colleagues in Germany [305] – Obtained after passages of the CVA strain in chicken embryo fibroblasts (CEFs), roughly 15% of its genome was lost due to six big deletions and many smaller alterations [306,307]. MVA is unable to reproduce in most mammalian cells in culture, including human cells and lacks several immunomodulators and host range genes seen in other VACV strains [308,309] – The prime-boost Imvamune group had evidence of viral excretion from the throats of two of six animals after challenge [304]
New York VACV (NYVAC)	NYVAC, like MVA, is unable to replicate in human cells. However, unlike MVA, expression of several late NYVAC proteins is restricted due to a translational block [310]. Despite being attenuated, NYVAC still elicits a strong immunological response [311,312]	Derived from a plaque-cloned isolate of the Copenhagen vaccine strain by deletion of 18 ORFs from the viral genome. Among the deleted ORFs, two genes are involved in nucleotide metabolism, the thymidine kinase (ORF J2R) and the large subunit of the ribonucleotide reductase (ORF I4L) [313]
DNA vaccine consisting of four VACV genes (L1R, A27L, A33R and B5R)	– After an otherwise deadly challenge with MPX, rhesus macaques treated with a DNA vaccine were protected from severe illness. Vaccinated animals with a single gene (L1R) that encodes a neutralizing antibody target developed severe illness but survived. This is the first proof that vaccinating against SPX and MPX with a subunit vaccine is possible [289] – Animals given only DNA did not have high titre Abs, had numerous skin lesions after being challenged and died similarly to placebo controls. Animals given proteins had moderate to severe illness (20-155 skin lesions) but lived. Individuals inoculated with DNA and then boosted with proteins had minimal illness, with 15 or fewer lesions that resolved in a matter of days [292]	
Dryvax-derived ACAM2000 (second-generation smallpox vaccines)	– ACAM2000 did not provide protection against severe MPX disease or mortality when given after exposure [261] – Prairie dogs were protected to some extent from the 2 LD 50 challenge of MPX disease, but not from the 170 LD 5 challenge. In the 2 LD 50 challenge, giving the vaccine one day after exposure was more effective than giving it three days later for Imvamune, while ACAM2000 was equally efficacious at both post-exposure vaccination times [314]	– Treatment with tecovirimat alone or in combination with ACAM2000 after exposure gave complete protection [261] – The only SPX vaccine now available in the United States that has been licensed by the FDA [294]
– Elstree-RIVM and Elstree-BN	No substantial reduction in mortality was seen in monkeys vaccinated 24 h after MPX infection with a typical human dose of Elstree-RIVM. All surviving animals had virus-specific blood antibodies and antiviral T cells after antiviral medication was stopped 13 days after infection [269]	– Elstree-RIVM is a first-generation SPX vaccine produced on calf skins [293] – Elstree-BN is a second-generation vaccine, passaged and produced on CEFs to further attenuate the virus and to make a better-defined vaccine preparation that does not depend on the use of calves [293]

## Conclusions

Epidemiological research to control the current MPX outbreak should consider the source of infection and all transmission routes. The current therapeutic regimens and vaccines that have been shown to be effective against SPX offer new approaches to the clinical treatment and prevention of MPX, which is essential in control of the current outbreak. Although management of MPX infection is still limited to treating secondary bacterial infections, reducing the symptoms and giving supportive care, FDA-approved anti-SPX treatments (CMX001 and ST-246) have shown efficacy against MPX. In this emergency situation, testing treatments with proven antiviral activities against VARV or other poxviruses may promote the pace of development of anti-MPXV drugs. Moreover, the world should turn the obstacles faced during outbreaks of infectious diseases that have emerged in the past into lessons to control the current outbreak by active cooperation, which is important in global efforts to combat the outbreak.

## Supplementary Material

Supplemental Material
