# Supplementary material for: Comprehensive literature review of monkeypox
Source: Emerg Microbes Infect. 2022 Nov 4;11(1):2600–31. doi: 10.1080/22221751.2022.2132882 (PMC9627636; doi:10.1080/22221751.2022.2132882)
Supplement: Supplemental Material [file TEMI_A_2132882_SM6352.docx]

Supplemental Table 1. Details of MPXV sequences detected in the 2022 outbreak.

| Country (number of detected sequences) | GenBank accession numbers |
| --- | --- |
| Spain (8) | ON631241.1ON782055.1; ON782054.1; ON745225.1; ON720849.1; ON720848.1; ON609725.2; ON622718.1 |
| Brazil (1) | ON751962 |
| Slovenia (6) | ON631241.1; ON754987.1; ON754986.1; ON754985.1; ON754984.1; ON609725.2 |
| Italy (7) | ON780017.1; ON780016.1; ON745215.1; ON644344.1; ON622721.1; ON614676.1 |
| France (10) | KJ642616.1; ON602722.2; ON755040.1; ON755039.1; ON622722.2; MF437052.1; MF437051.1; AF375110.1; JF799095.1; JF792045.1 |
| The Netherlands (3) | KJ642614.1; ON615424.1; AF375113.1 |
| USA (2) | ON563414.3; NC_063383.1 |
| Germany (50) | ON755256.1; ON755255.1; ON755254.1; ON755253.1; ON755252.1; ON755251.1; ON755250.1; ON755249.1; ON755248.1; ON755247.1; ON755246.1; ON755245.1; ON755244.1; ON755243.1; ON755242.1; ON755241.1; ON755240.1; ON755239.1; ON755238.1; ON755237.1; ON755236.1; ON755235.1; ON755234.1; ON755233.1; ON755232.1; ON755231.1; ON682270.2; ON682269.3; ON682268.2; ON682267.2; ON682266.2; ON682265.3; ON682264.3; ON682263.3; ON694342.1; ON694340.1; ON694339.1; ON694338.1; ON694341.1; ON694337.1; ON694336.1; ON694335.1; ON694334.1; ON694333.1; ON694332.1; ON694331.1; ON694330.1; ON694329.1; ON637939.1; ON637938.1 |
| Portugal (28) | ON585038.1; ON585037.1; ON585036.1; ON585035.1; ON585034.1; ON585033.1; ON585032.1; ON585031.1; ON585030.1; ON585029.1; ON649725.1; ON649724.1; ON649723.1; ON649722.1; ON649721.1; ON649720.1; ON649719.1; ON649718.1; ON649717.1; ON649716.1; ON649715.1; ON649714.1; ON649713.1; ON649712.1; ON649711.1; ON649710.1; ON649709.1; ON649708.1 |
| UK (2) | ON622713.1; ON622712.1 |
